# QSAR Modeling, Molecular Docking and Cytotoxic Evaluation for Novel Oxidovanadium(IV) Complexes as Colon Anticancer Agents

**DOI:** 10.3390/molecules27030649

**Published:** 2022-01-19

**Authors:** Fatimah Y. Alomari, Abeer A. Sharfalddin, Magda H. Abdellattif, Doaa Domyati, Amal S. Basaleh, Mostafa A. Hussien

**Affiliations:** 1 Chemistry Department, College of Science, Imam Abdulrahman Bin Faisal University, P.O. Box 76971, Dammam 31441, Saudi Arabia; falumary@stu.kau.edu.sa; 2 Department of Chemistry, Faculty of Science, King Abdulaziz University, P.O. Box 80203, Jeddah 21589, Saudi Arabia; asharafaldin@stu.kau.edu.sa (A.A.S.); abasaleh@kau.edu.sa (A.S.B.); 3 Department of Chemistry, College of Science, Taif University, Al-Haweiah, P.O. Box 11099, Taif 21944, Saudi Arabia; m.hasan@tu.edu.sa; 4 Department of Chemistry, College of Science, University of Jeddah, P.O. Box 80327, Jeddah 21589, Saudi Arabia; dmdomyati@uj.edu.sa; 5 Department of Chemistry, Faculty of Science, Port Said University, Port Said 42521, Egypt

**Keywords:** oxidovanadium(IV) complexes, DFT modeling, DNA binding, docking, QSAR study

## Abstract

Four new drug-based oxidovanadium (IV) complexes were synthesized and characterized by various spectral techniques, including molar conductance, magnetic measurements, and thermogravimetric analysis. Moreover, optimal structures geometry for all syntheses was obtained by the Gaussian09 program via the DFT/B3LYP method and showed that all of the metal complexes adopted a square-pyramidal structure. The essential parameters, electrophilicity (ω) value and expression for the maximum charge that an electrophile molecule may accept (ΔN_max_) showed the practical biological potency of [VO(CTZ)_2_] 2H_2_O. The complexes were also evaluated for their propensity to bind to DNA through UV–vis absorption titration. The result revealed a high binding ability of the [VO(CTZ)_2_] 2H_2_O complex with K_b_ = 1.40 × 10⁶ M^−1^. Furthermore, molecular docking was carried out to study the behavior of the VO (II) complexes towards colon cancer cell (3IG7) protein. A quantitative structure–activity relationship (QSAR) study was also implemented for the newly synthesized compounds. The results of validation indicate that the generated QSAR model possessed a high predictive power (R^2^ = 0.97). Within the investigated series, the [VO(CTZ)_2_] 2H_2_O complex showed the greatest potential the most selective compound comparing to the stander chemotherapy drug.

## 1. Introduction

Cancer is one of the leading causes of death, the fatal characteristic of which is uncontrollable and irregular growth. In cancer, some cells of the diseased part start to grow abnormally and can metastasize to a different part of the body [1]. Among the different types of cancer, colon cancer represents the fourth most common type of cancer and the fifth major cause of cancer-associated death [1,2]. Thus, there is a need to effect corrective measures to reduce these numbers, such as finding influential anticancer agents and achieving early screening of the cancer cell, which is dependent on the patient and the cancer type [3].

In the past few years, there has been increased interest in developing additional transition metal compounds as anticancer drugs due to their lower toxicity than platinum-based agents [4]. Among the transitional metals, vanadium complexes have been studied and have shown practical activity towards various tumor cell types [5]. This finding suggests that vanadium complexes have beneficial therapeutic properties that could be extended to treat common types of cancer [5,6,7,8]. The most practical role of these anti-cancer agents is the checking and correcting of the division of cancer cells before they continue their amorphous growth [9,10,11]. There are massive studies that have explored the introduction of the metal ion to medication drugs to enhance their efficiency for pharmacological applications, as well as for several other unique therapeutic opportunities [12,13,14]. The new metal-based drugs have lower toxicity and high lipophilicity to penetrate the cell, and greater absorbance and stability than the free drugs [15]. Oxidovanadium(IV) complexes of interest are given in the work of Biswal and his colleagues, who synthesized three novel water-soluble oxidovanadium(IV) compounds [16], and two of the obtained complexes showed significant anticancer activities against the human hepatic carcinoma cell line Hep3B, which could be considered as a candidate for further studies. In this light, the current work aims to conduct descriptive and predictive research concerning anticancer activity for a new series of oxidovanadium(IV) complexes. Four drugs have an important medicinal feature, such as antibacterial (sulfonamide), antihistamine (cetirizine), antithyroid (carbimazole), and anti-inflammatory (Lornoxicam) activities, and have different donating atoms to react with oxidovanadium(IV) and to test their efficiency.

One of the approaches to accelerating the drug design process through the compounds’ advantages is through computer-aided drug design (CADD) [17,18]. Two approaches that are often used in CADD are quantitative structure–activity relationships (QSAR) and molecular docking (MD). The basis of QSAR is classification and regression techniques that can be characterized as a strategy attempting to foretell the characteristics, reactivity, and activity of a fuzzy set of molecules, based on the investigation of an equation that connects the molecular configurations to their specific measured property and activity [19,20]. Currently, QSAR is one of the most important methodologies in pharmacy and chemistry [21,22] used to develop mathematical models to identify statistically noteworthy correlations between toxicological, biological, binary, categorical, and continuous properties, and chemical structure. Additionally, molecular docking is based on the binding characteristics of the target and ligand. It creates various potential adduct structures, clustered and classified together through software that scores them. Examples of the areas where molecular docking is utilized include the design of drugs and in understanding the molecular interaction of drugs [23,24,25].

Therefore, an in silico QSAR study was performed to rationalize the structure–activity relationship using a molecular operating environment (MOE) for the studied metal complexes. This method can reduce the experimental time and effort required to examine all of the investigated compounds and focus on the promising molecules to further investigate by in vitro study. Additionally, the molecular docking technique was also carried out for all oxidovanadium(IV) compounds to exhibit the binding modes and interactions between the compounds and the colon cancer protein. An in vitro assay was used to confirm the in-silico simulation results.

## 2. Results and Discussion

### 2.1. Physical Properties

All synthesized VO(II) complexes were stable at room temperature and virtually insoluble in water and most organic solvents, but were soluble in some solvents, including DMSO and DMF. The molar conductance values of [VO(SO_4_)(CBZ)] 8H_2_O, [VO(CTZ)_2_] 2H_2_O and [VO(SO_4_)(SCZ)] 7H_2_O complexes at 1 × 10^−3^ M in DMSO solution were 7.47, 8.85, and 17.72 Ω^−1^ mol^−1^ cm^2^, respectively, as given in Table 1**.** These low values indicate that the complexes were nonelectrolytes. At the same time, the [VO(LOR)_2_] SO_4_ complex gave a value of 69.85 Ω^−1^ cm^2^ mol^−1^, reflecting the formation of a cationic molecule or the existence of a sulfate group in the coordination sphere [26,27].

**Figure 1:**
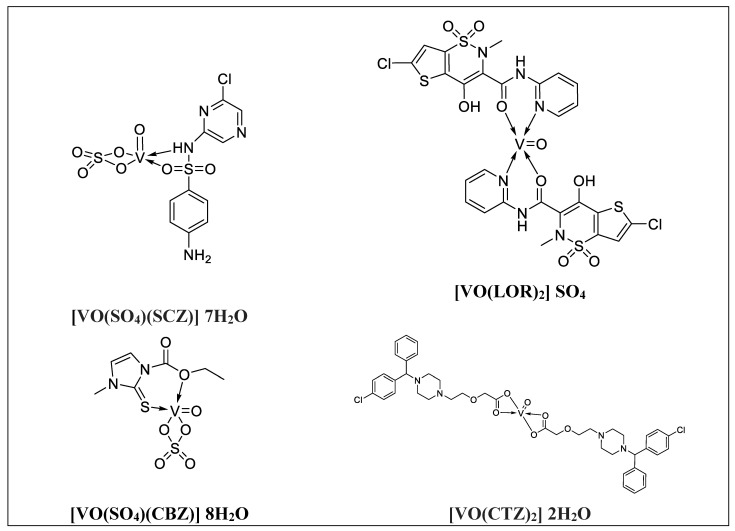
The suggested structures for oxidovanadium(IV) complexes.

The proposed complex structures were confirmed by elemental analysis, conductometrics, and physical measurements. The elemental analyses and physical properties of the ligands and their vanadium complexes are summarized in Table 1 and the suggested structure is shown in Figure 1.

### 2.2. The Molar Ratio of the Complexes

The stoichiometric environments around the oxidovanadium(IV) complexes were identified using a molar ratio technique [28]. The absorbance values were plotted toward the ratio of [M]/([M]+[L]) and are shown in Figure S1. The reflection line upon increasing the ligand concentration was around 0.5, which indicates the formation of [VO(SO_4_)(CBZ)] 8H_2_O and [VO(SO_4_)_2_(SCZ)] 7H_2_O complexes with 1:1 (M:L). In contrast, [VO(CTZ)_2_] 2H_2_O and [VO(LOR)_2_] SO_4_ complexes gave a reflection line around 0.33, which suggested a molar ratio of 1:2 (M:L).

### 2.3. Infrared Spectra (IR)

**Figure 2:**
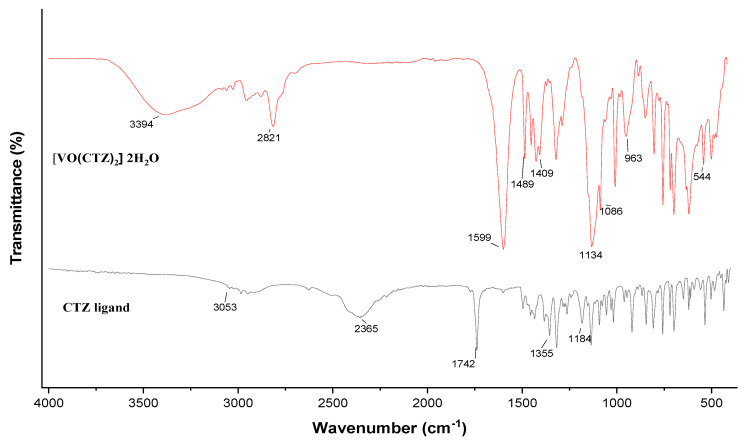
A comparison of the IR spectra of free CTZ ligand and its oxidovanadium(IV) complex in the range 4000–500 cm^−1^.

The IR spectra of the free ligands and their complexes are shown in Figure 2 and Figure S3. The main IR bands and their assignments are listed in Table 2. The IR spectra of complexes showed broad bands in the region of 3450–3540 cm^−1^, corresponding to the OH stretching vibrations of the lattice water molecules. The IR spectrum of the free CBZ ligand showed three bands for the ester group, at 1677, 1228, and 1096 cm^−1^ for the ν (C=O) and two ν (C–O) stretches, respectively. No notable change was seen in the ν(C=O) band, indicating that it did not coordinate with the vanadyl ion. Moreover, there was a noticeable shift in the ester oxygen bands to 1234 and 1107 cm^−1^, and these low frequencies confirmed the coordination of the ester oxygen with the vanadium ion [15]. The band at 1271 cm^−1^ related to the ν (C=S) vibration shifted to 1279 cm^−1^, which was a strong indication that the sulfur atom contributed to the complex was a donor [15]. A new band observed in the range 1310–1276 cm^−1^ was attributed to ν_as_(SO_4_) and ν_s_(SO_4_) bands.

In the [VO(CBZ)_2_] 2H_2_O complex, it is possible to observe the disappearance of the absorption band in the region around 1742 cm^−1^ assigned to the free carboxylic acid of CBZ, which indicated the formation of a bond between the vanadium ion and the carboxylate oxygen. The band corresponding to ν(C=O) was replaced by two bands at 1599 and 1409 cm^−1^, assigned to antisymmetric ν_asym_(COO^−^), and symmetric ν_sym_(COO^−^), respectively. The difference Δν [Δν = ν_asym_(COO^−^)–ν_sym_(COO^−^)] is a useful tool for determining the coordination mode of the carboxylate ligands [29]. The calculated value was in the range = 189 cm^−1^, which indicated a bidentate coordination mode for the cetirizine ligand [30,31].

The free LOR ligand showed bands in the ranges 1647 and 1591 cm^−1^, which were assigned to ν(C=O), and the ν(C=N) stretching frequency shifted to 1668 and 1621 cm^−1^, respectively, in the metal complex, indicating the involvement of the nitrogen of the pyridine ring and the oxygen of the amide group in the complex formation.

The IR spectrum of the free SCZ ligand showed a band corresponding to the ν(N-H) mode of the sulfonamide group and appeared at 3282 cm^−1^. This band shifted from the main position due to hydration or may have coordinated with metal [32,33]. The band at 1670 cm^−1^ assigned to ν(C=N) remained in complex, indicating the non-involvement of this group in the coordination sphere [34]. The ν(S-N) band at 1097 cm^−1^ shifted to 1105 cm^−1^, and hence confirmed the coordination of the metal center to the nitrogen of the sulfonamide. Two new bands observed in the [VO(SO_4_)(SCZ)] 7H_2_O spectrum at 1145–1011 cm^−1^ were assigned to ν_as_(SO_4_) and ν_s_(SO_4_) bands, in bidentate mode.

All four complexes exhibited a band at 955–960 cm^−1^, which can be ascribed to ν(V=O) stretching frequency. The band position of ν(V=O) agrees with the square-pyramidal structure [34]. Moreover, the bands in the regions of 560–580, 635–655, and 422 cm^−1^ may be assigned to (V–O), (V–N), and (V–S) stretching frequencies, respectively.

### 2.4. Electronic Spectroscopic and Magnetic Susceptibility

**Figure 3:**
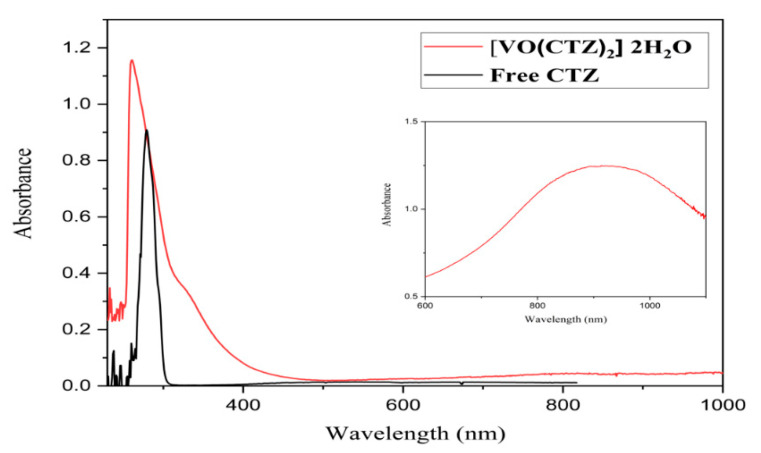
Electronic absorption spectrum of free CTZ ligand and its metal complex (inset shows the d-d transition the metal complex).

The UV–Vis spectroscopic analysis was performed for the ligands and their VO(II) complexes in DMSO in the range of 200–1000 nm and is shown in Figure 3 and Figure S4. The transitions in the free ligands attributed to π–π* and n–π* at the 260–280 and 311–373 nm regions in the UV spectra of the complexes shifted to low or high frequencies, confirming the coordination of the ligands to vanadyl ion. The observed weak-intensity bands in the range 850–900 nm in the metal complexes were assumed to metal-centered d–d transitions.

All synthesized complexes had magnetic moment values between 1.76 and 1.74 B.M. at 25 °C in accordance with the 3d^1^ electronic configuration of the V^IV^O^2+^ moiety. These values are compatible with the reported values for the square-pyramidal geometry [35]. The magnetic moment values and the electronic transition data for the compounds are presented in Table 3.

### 2.5. Electron Paramagnetic Resonance Spectra (EPR)

**Figure 4:**
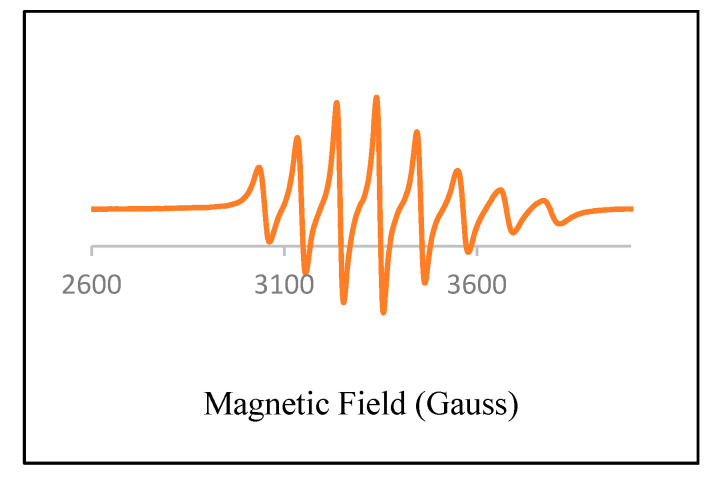
EPR spectrum of [VO(CTZ)_2_] 2H_2_O in DMF at room temperature.

The X-band EPR spectra for the oxidovanadium(IV) complexes were acquired in DMF at room temperature. The stable isotope, ^51^V, of the vanadium (IV) ion carries electron and nuclear spins of S = 1/2 and I = 7/2, respectively. The EPR spectra of oxidovanadium(IV) complexes display eight absorptions due to the coupling of the vanadyl unpaired electron with the vanadium nuclear spin moment. These data suggest that just single mononuclear vanadium (IV) species containing a lone unpaired electron exist in the examined sample solution. The EPR parameters g and A values are presented in Table 4. The calculated values are consistent with a square-pyramidal structure [36,37]. Overall, the g values were less than those for the free electron: g_e_ = 2.002. This phenomenon could be a consequence of the spin–orbit interplay of the ground state, d_xy_ level, with the low-lying excited states [38]. The EPR spectra for oxidovanadium(IV) complexes are shown in Figure 4 and Figure S5.

### 2.6. Thermal Analysis (TGA)

**Figure 5:**
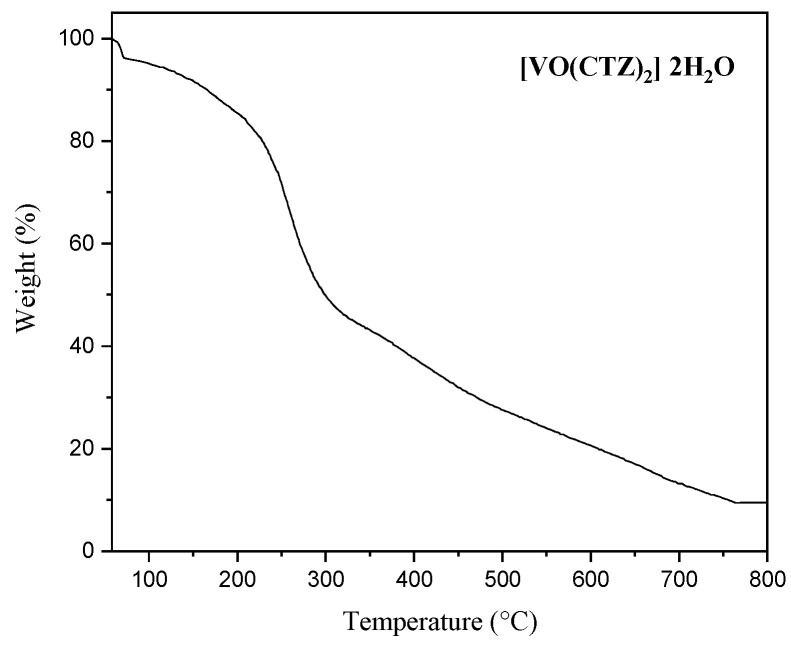
The TGA curve decomposition in the range 25–800 °C of [VO(CTZ)_2_] 2H_2_O complex.

Thermogravimetric studies of all complexes showed two decomposition stages. The obtained curves are displayed in Figure 5 and Figure S6. The initial step for [VO(SO_4_)(CBZ)] 8H_2_O occurring between a temperature of 25 °C and 218 °C corresponded to a loss of C_4_H7N_2_O_3_S and eight water molecules of hydration with weight loss 55.19% The second stage occurred within the temperature of 218 °C to 800 °C was related to loss of C_5_H_3_O_2_S with 27.55% mass loss, followed by the formation of VO_2_ as a residue. 

The first decomposition step of [VO(CTZ)_2_] 2H_2_O complex was removed two lattice water molecules in between 55 and 100 °C with weight loss 4.09%. The organic decomposition began at ~120 °C with 86.46% The residue consisted of VO_2_ with a weight loss of 9.44%.

In contrast, the [VO(LOR)_2_] SO_4_ complex did not show a weight loss up to 119 °C corresponds to the absence of lattice water molecules. Therefore, the weight loss in the range 119–341 °C has corresponded to the loss of C_7_H_6_C_l2_O_10_S_2_ with a total weight loss of 42.36%. At the same time, a weight loss of 48.26% within the temperature range 341–552 °C is assigned to losing C_19_H_12_N_6_OS_3_ and forming VO_2_; as a residue with a final weight of 9.38%.

The complex [VO(SO_4_)(SCZ)] 7H_2_O lost C_6_H_8_N_2_O_4_S_2_ and seven hydrated water molecules with a weight loss of 69.37%, then followed by losing 16.22% of the mass in a temperature range of 232–998 °C which assigned to C_4_HN_2_O. The final product is metal residue with a total mass loss amounts to 14.40%. The TGA data for the VO(II) complexes present in Table S2.

### 2.7. Kinetic Analysis for Thermogravimetric Data

The kinetic parameters were calculated using Coast–Redfern (CR) and Horowitz–Metzger (HM) methods. The kinetic parameters were obtained via equations presented in previous work [39] {Sharfalddin AA, 2021 #63} and showed in Table S3. The values of E_a_ indicate that the complexes are extremely stable. The values of ΔS are negative and indicated slower thermal decomposition than the typical reaction [40]. Positive values of ΔH and ΔG indicate that all decomposition steps are endothermic and non-spontaneous processes. The high values of ΔG for each stage are due to the increase in TΔS values from one step to the next. The CR and HM curves for the oxidovanadium(IV) complexes are represented in Table S4.

### 2.8. Molecular Modeling of Investigated Compounds

#### 2.8.1. Geometry Description

The results obtained from elemental analysis, molar ratio, molar conductance, spectroscopic techniques, and thermal studies were used to build the input files for all synthesized oxidovanadium(IV) complexes and free ligands, running the jobs using the DFT method through the 6–31G and LANL2DZ basis sets. The best-optimized geometries and the bond length of V=O were visualized by Gauss View and are presented in Table 5 and Figure S7. The formed structure of the metal complexes is square-pyramidal geometry with negligible distortion. The bond length around the metal ion was in the range 1.95–2.60 Aº between the donating atoms and the vanadyl ion, which showed a range of small and medium ionic character (bond length > 2 Å) [41]. On the other hand, there was a direct correlation between the length of a metal–oxygen bond and its stretching frequency. Thus, we can directly determine the metal–oxygen bond length from the measurement of stretching frequencies, calculated by the below equation [42,43]:

v = 21,349 exp(−1.9176 R (Å))

where v is the stretching frequency for V=O in cm^−1^, and R is the V=O bond length in angstroms. The stretching frequencies and the respective metal−oxygen bond lengths have an inverse relationship. This means that a higher stretching frequency corresponds to a shorter metal−oxygen bond length. Therefore, it is possible to utilize IR spectra information to compute the V=O bond length.

A comparison of the experimental frequencies and the extracted theoretical data is presented in Table 6. The obtained experimental bond length by the above equation for the investigated compounds was in the range 1.603–1.616 Å and correlated with the theoretical data. 

The highest occupied molecular orbital (HOMO) and lowest unoccupied molecular orbital (LUMO) represent the nucleophilicity of the molecule (electron donating) and the electrophilicity (electron accepting) of the molecule, respectively. Thus, the images and energy values of HOMO and LUMO were extracted in the ground state to study the electronic characteristics of molecular systems for the ligand and around the metal center. Table S5 compares the molecular orbital diagrams between the ligand and the oxidovanadium(IV) complexes. Interestingly, both HOMO and LUMO orbitals in the free CTZ were concentrated in the ((4-chlorophenyl)(phenyl)methyl)-4-methyl piperazine part. After complexing with a metal ion, the HOMO orbital appeared prolonged over a 1-((4-chlorophenyl) (phenyl)methyl)-4-methyl piperazine part of the complex, while the LUMO orbital was located around the central metal. In contrast, a free LOR ligand showed its HOMO over the whole molecule, except the pyridine ring, while the LUMO level presented in 2-chlorothiophene. These locations of the HOMO and LUMO were maintained after reaction with VO(II).

#### 2.8.2. Global Reactivity Descriptors

Using details from these molecular orbital compositions and energy levels, global reactivity descriptors were calculated as the following: energy gap (∆E = E_gap_ = E_LUMO_ −E_HOMO_), absolute electronegativities (χ = −E_HOMO_ + E_LUMO_/2), absolute hardness (η = E_LUMO_ − E_HOMO_/2), chemical potentials (μ = −w), global softness (S = 1/2η), and global electrophilicity (ω = π2/2η) [39] and are shown in Table 7.

The energy gap, E_gap_, shows a practical guide for the reactivity of the compound [39]. Decreasing the level between the HOMO and LUMO orbitals led to lowering the energy gap E_gap_, thus enhancing the transition of the electrons and the softness of the complex. We can observe from Table 7 that complexing the ligand significantly minimized the energy gap of the complexes. The oxidovanadium(IV) of LOR has the lowest energy gap among the investigated complexes. Moreover, the global softness (S) has moderate values, indicating the minor characteristics of synthesized compounds. The electrophilicity (ω) is an expression of the ability to obtain electronic charge from other molecules in the environment, increasing the biological activity descriptor. The highest value appeared with the VO–CTZ complex (5.03 eV), followed by VO–SCZ and VO–CBZ, which reveal their biological potency. The ΔN_max_ is an expression of the maximum charge an electrophile may accept from the environment [44]. We could arrange the metal complexes depending on this value in descending order, VO–CTZ > VO–LOR > VO–SCZ > VO–CBZ, which represents the capacity of VO–CTZ to be an anticancer agent.

#### 2.8.3. DNA Binding Study

**Figure 6:**
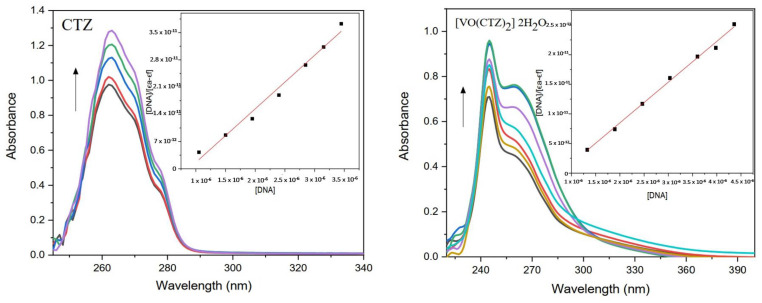
Absorption spectra of CTZ and its metal complex in the presence of increasing DNA concentration (arrow indicates changes with increasing DNA concentration).

A DNA-binding assay is an effective technique for investigating a new compound’s biological activity by studying its ability to interfere with DNA replication and transcription [45,46]. Absorption titration is a standard and easy approach to determine the interaction mode and the nature of DNA binding [47,48]. The spectral parameters for the interaction of the tested complexes with DNA are presented in Table 8, whereas the chromic absorption spectra are presented in Figure 6 and Figure S8.

All compounds showed an increasing hyperchromic shift with increasing CT–DNA concentration, except the LOR ligand, which showed a hypochromic shift. The hyperchromic effect implies that the ligand and its complex bind to the external of the major or minor grooves of DNA via electrostatic binding [49,50]. Moreover, hyperchromic observation indicates the alterations in the structure of the genetic material following engagement with the complex [45]. Otherwise, the hypochromic effect is an intercalative reaction between the aromatic part of the compound with DNA bases [51,52]. Obviously, the complex [VO(CTZ)_2_] 2H_2_O showed the highest K_b_ value (1.40 × 10^6^), which implies that the compound has a better binding ability compared with other compounds.

#### 2.8.4. Molecular Docking

Molecular docking analysis plays a significant part in investigating the complexation of a ligand at the active site of a receptor. Thus, molecular docking analysis was conducted to elucidate how the free ligands and the oxidovanadium(IV) complex interact with the investigated protein. The dock procedures were evaluated against colon cancer (3IG7), which was chosen based on previous research suggesting its inhabitation as colon cancer therapy [53,54]. The binding energy scores for free ligands and their synthesized oxidovanadium(IV) compounds are presented in Table 9. The oxidovanadium(IV) complexes initially exhibited more potent binding energy than their free ligands against 3IG7 protein. They indicated that the modified vanadyl complexes enhance the binding affinity of the free ligands toward the examined protein. At the same time, the [VO(CTZ)_2_] 2H_2_O complex has the highest binding with the lowest negative scoring of −9.81 KJ mol^−1^, which is related to the π-acceptor interaction between a six-membered ring containing a nitrogen atom in ASN 132 amino acid with an energy score of −1.6 kcal mol^−1^. The docking scores for all oxidovanadium(IV)) complexes and free ligands are shown in Table 10. The interactions between all complexes and 3IG7 proteins are represented in Table S6.

#### 2.8.5. QSAR Study

The common applications of QSAR modeling include the evaluation of the structural characteristics of a compound, and the design and prediction of the activities of new compounds. Therefore, MOE software was applied to develop a QSAR model of oxidovanadium(IV) complexes. Several descriptors, including ASA_P, ASA_H, h_pkp, and dipole moment, were utilized to display the predicted IC_50_ values. These were close to the actual IC_50_ values for the training set, as presented in Figure S2. Out of the MLR analysis, the model exhibited a regression coefficient (R) of 0.9882 and coefficient of determination (R^2^) of 0.9766, thus explaining the 97.66% IC_50_ activity. The R and R^2^ figures portray the accuracy of the QSAR model. 

**Figure 7:**
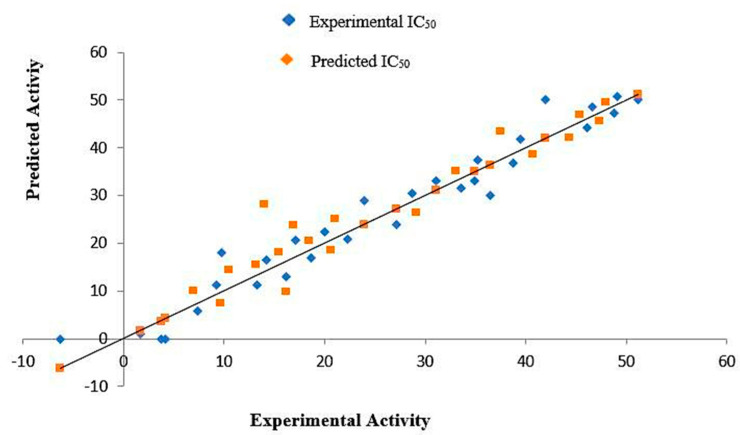
MLR correlation between Predicted anticancer activities of “IC_50_” and experimental values of training set.

The plot of the relationship between the predicted values obtained from MLR and the experimental data is shown in Figure 7. Some predicted and experimental values exhibit a close correlation, and residual (RE) values were close to zero, indicating that the predictions are reliable, as shown in Table S7. According to the results, the approach was recorded as the QSAR model and applied to predict the compound activities of the test data set. Based on the QSAR model, a fit was used to obtain the predicted activity (PRED IC_50_) data of the synthesized oxidovanadium(IV) complexes and their free ligands (testing set), and the results are presented in Table 11. The [VO(CTZ)_2_] 2H_2_O complex exhibited the best predicted activity (PRED IC_50_ = 1.45 µM), which indicated that the complex was more effective against colon cancer. On the other hand, the complexes [VO(SO_4_)(SCZ)] 7H_2_O and [VO(SO_4_)(CBZ)] 8H_2_O showed the lowest predicted activities against colon cancer. The other members showed a moderate predicted activity, which ranged between PRED IC_50_ values of 2.61 and 32.87 µM. Interestingly, the predicted IC_50_ values of [VO(SO_4_)(CBZ)] 8H_2_O, [VO(CTZ)_2_] 2H_2_O, and [VO(LOR)_2_] SO_4_ complexes were higher than their free ligands, while the other complexes were less active than their free ligands.

### 2.9. Anticancer Study

Based on the results obtained from the QSAR and docking studies, the [VO(CTZ)_2_] 2H_2_O complex was selected for examination against the human colon cancer cell line (HCT116) to evaluate its anticancer properties. The cytotoxicity of cisplatin against the HCT116 cell line was used to benchmark the anticancer activity of the oxidovanadium(IV) complex. The results are listed in Table 12. The IC_50_ for [VO(CTZ)_2_] 2H_2_O was 2.11 μM, indicating potent anticancer activity compared with the standard chemotherapy drug and with cisplatin, which has an IC_50_ of 44.13 μM. Furthermore, the obtained value strongly correlates with the predicted score from the QSAR equation (PRED IC_50_ = 1.45 µM). The selectivity of the compound showed low toxicity toward normal cell line (LLC-MK2) with an IC_50_ value of 649.8 µM which illustrated good selectivity and practical activity.

Comparing this result with previous work, the Schiff base oxidovanadium(IV) complex were tested against the colon cancer cell line (HCT116), with an IC_50_ = 2.39 µM. Hence, the [VO(CTZ)_2_] 2H_2_O has higher potency and could be considered as an anticancer agent [55].

## 3. Materials and Methods

### 3.1. Materials and Reagent

VOSO_4_ was supplied from Sigma Aldrich. Carbimazole (CBZ) and Sulfaclozine (SCZ) were obtained from Alibaba (China). Lornoxicam (LOR) and Cetirizine (CTZ) were obtained from Tabuk Pharmaceuticals (Tabuk, Saudi Arabia). Calf thymus DNA (CT-DNA) from SIGMA type 1A36 was used for the DNA studies. All the other solvents were obtained from Sigma-Aldrich (St. Louis, MO, USA) and were used as received. 

### 3.2. Apparatuses

The element’s content (CHN) was observed using Perkin-Elmer 2400 CHN Elemental Analyzer instrument. Melting points were measured by MPA100–Automated melting point system. The conductivity measurements are determined using a conductor TDA meter at room temperature in 10^−3^ M DMSO solutions. IR spectra were recorded using solid samples on a PerkinElmer Spectrum 100 instrument (Waltham, MA, USA); peaks are reported over the range 4000–400 cm^−1^. The electronic absorption spectra were determined in the DMSO solution for the free ligands and their complexes using a MultiSpec-1501 UV–Vis spectrophotometer with 1 cm quartz cells; bands in the range 200–1100 nm. The X-band EPR spectra for all the complexes are recorded as in DMF solution at room temperature from the continuous wave Bruker EMX PLUS spectrometer (Bruker BioSpin, Rheinstetten, Germany) and collected with Bruker Xenon software with DPPH standard (υ = 9.440103 GHz). Thermal analysis was carried out for the complexes under a nitrogen atmosphere, a constant heating rate was applied by 10 °C min^−1^ at a temperature range from 25 to 1000 °C using the PerkinElmer TGA system.

### 3.3. Molar Ratio Method

A standard solution of the metal ion was consistent at 0.72 × 10^−4^ M and divided between seven volumetric flasks by pipette into the volumes of 0, 1, 2, 3, 4, 5, and 6 mL. Then, various ligand concentrations were added in 6, 5, 4, 3, 2, 1, and 0 mL volumes, as presented in Table S1. All measurements were performed in the 200 to 500 nm spectrum [28,56].

### 3.4. Synthesis of VO(II) Complexes

All the metal complexes were prepared by adding a 3 mL solution of VOSO_4_.5H_2_O (1 mol) to 25 mL of ethanol to dissolve the free ligand, according to the stoichiometric amount extracted from the molar ratio experiment. The obtained mixture was refluxed for 2 h and the formed precipitate was filtered, collected, and then washed several times with diethyl ether, before being oven-dried overnight at 55 °C.

**[VO(SO_4_)(CBZ)] 8H_2_O**: Yield 63%, m.p. 125 °C. IR (KBr, cm^−1^): 3275 ν(OH_2_); 1310 νas(SO_2_); 1035 νs(SO_2_); 1234, 1107 ν(C-O); 1081 ν(C-Cl); 980 ν(V=O); 596 ν(V-O); 422 ν(V-S). Elemental analysis of C7H26N2O15S2V (%): Found: C 17.04; H 5.31; N 5.68; Anal. Calc. (%): C 17.25; H 5.32; N 5.60.

**[VO(CTZ)_2_] 2H_2_O**: Yield 89%, m.p. 134 °C. IR (cm^−1^): 3394 ν(OH_2_); 1599 ν_asym_(COO^−^); 1409 ν_sym_(COO^−^); 963 ν(V=O); 544 ν(V-O). Elemental analysis of C_42_H_52_Cl_2_N_4_O_9_V (%): Found: C 57.41; H 5.96; N 6.38; Anal. Calc. (%): C 57.43; H 5.97; N 6.41.

**[VO(LOR)_2_] SO_4_**: Yield 68%, m.p. 124 °C. IR (cm^−1^): 3348 ν(OH); 1668 ν(CONH); 1621 ν(C=N); 1325 ν_asym_(SO_2_); 1034 ν_sym_(SO_2_); 968 ν(V=O); 521 ν(V-O); 469 ν(V-N). Elemental analysis of C26H20Cl2N6O11S5V (%): Found: C 35.71; H 2.31; N 9.61; Anal. Calc. (%): C 35.73; H 2.30; N 9.54.

**[VO(SO_4_)(SCZ)] 7H_2_O**: Yield 61%, m.p. 209 °C. IR (cm^−1^): 3445 ν(OH_2_); 3282 ν(N-H); 1670 ν(C=N); 1271 ν_asym_(SO_2_); 1033 ν_sym_(SO_2_); 1105 ν(S-N); 968 ν(V=O); 589 ν(V-O); 424 ν(V-N). Elemental analysis of C_10_H_23_N_4_O_14_S_2_V (%): Found: C 20.93; H 4.04; N 9.76; Anal. Calc. (%): C 30.10; H 4.13; N 9.98.

### 3.5. Molecular Modeling Method

Theoretical calculations for the free ligands and their metal complexes were obtained using the Gaussian 09 package [57]. The geometry optimization was carried out by the density function theory method (DFT) with Becke’s three-parameter hybrid and Lee–Yang–Parr correlation functional method (B3LYP). The basis set (6–31G) was used for the organic molecules and the correlation-consistent basis set (LANL2DZ) as its preferred basis set for the metal complex [58]. The extracted data from the log files showed that the vibration frequency did not have any imaginary frequencies and that the molecule was a minimum on the potential energy surface. Gauss View software was used to visualize the fchk files and extract the E_LUMO_ and E_HOMO_ levels and energy orbitals to estimate the essential quantum parameters using the following equations: energy gap (E_gap_ = E_LUMO_ − E_HOMO_), absolute electronegativities (χ = −E_HOMO_ +E_LUMO_/2), absolute hardness (ɳ = E_LUMO_ − E_HOMO_/2), chemical potentials (μ = −χ), global softness (S = 1/2η), and global electrophilicity (ω = π2/2η) [41]. 

### 3.6. Molecular Docking Method

Molecular docking study utilized the MOE 2019.0102 program [59]. All optimized drugs and their metal complexes from the density functional theory method (DFT) were used in the docking process. Crystal configurations of the HCT116 (PDB code = 3ig7) were obtained from the Protein Data Bank (http://www.rcsb.org/pdb/ (accessed on 1 December 2021)) [60]. Any attached water or ligand cofactors, together with the water moieties in proximity to the protein, were deleted; a process followed by the correction of the protein structure, and then the addition of hydrogen atoms. An MMFF94x force field was used to allocate parameters and charges. Following the MOE synthesis of alpha-site spheres, the configured complex models were docked on the leading active site using MOE’s DOCK module [61,62,63]. A London dG scoring function was used for dock scoring within the MOE software, enhanced with the use of unconnected refinement techniques, and the superlative ten binding positions were directed for analysis to attain the optimum score. A database browser was recruited to perform a comparison of the docking poses with the ligand within the co-crystallized configuration and to generate their RMSD. The RMSD and the native ligand’s method of engagement within the receptor’s configuration were utilized as a standard docked model. The same protocol was utilized to dock the co-crystalline compound“N-{1-[cis-3-(acetylamino)cyclobutyl]-1H-imidazol-4-yl}-2-(4-methoxyphenyl)acetamide” which downloaded with the 3IG7 protein and treated as positive control while for negative control we used four different amino acids. For all docking result we get same docking site with the positive control and −7.30 kcal/mol with hydrogen bond of 2.5 Å. Where the negative control compounds do not show any interaction with very small docking score Tables S8 and S9.

### 3.7. QSAR Technique

#### 3.7.1. Data Set Used

A QSAR assessment was conducted on a set of 100 compounds to examine their colon anticancer activity. The compounds were grouped differently for testing and training. The literature shows that the imidazole derivative molecules and vanadyl complexes can be utilized to counter colon cancer activity. The structure and biological activities of the compounds obtained in the experiment are represented in Figure S2. Moreover, the synthesized vanadyl complexes and their ligands were used as test data. The training data set obtained from the biological activity (IC_50_) was used as the dependent variable, linearly correlated with the independent variables.

#### 3.7.2. Physiochemical Descriptors

A descriptor is a mathematical argument describing molecular properties according to biological activity and structure. The physicochemical descriptors calculated were total polar surface carrier (ASA_P), total hydrophobic surface (ASA_H), pK_b_ of the reaction to add a proton at pH 7 (h_pK_b_), and dipole moment (dipole).

#### 3.7.3. Development of Experimental Data Model

The QSAR model was formulated based on anti-cancer activities (IC_50_) (as the dependent variable), while descriptors were used as model fields. Multi-linear regression (MLR) analysis was applied to obtain a training data set. MLR depicts a direct linkage between the independent variable X, which represents the molecular descriptors, and the dependent variable Y, which represents the biological activity. In an MLR approach, the average value of Y is dependent on X. Below is an MLR equation that includes more than a single independent variable (descriptors), while maintaining one response variable (activity).

$$Y={\mathrm{bx}}_{1}+{\mathrm{bx}}_{2}+{\mathrm{bx}}_{3}+{\mathrm{bx}}_{4}+C$$

where Y is the dependent variable, b represents the regression coefficients of a varying x (independent variable), and C represents a regression constant.

### 3.8. Biological Applications

#### 3.8.1. CT-DNA Interaction

All studies with CT–DNA were carried out in Tris-HCl buffer solution (pH 7.4). The UV absorbance ratio (A260/A280) was 1.8–1.9, confirming that the DNA was free of protein. Electronic absorption spectra were obtained with a constant compound concentration at varying CT–DNA concentrations. The Benesi–Hildebrand equation (Equation (2)) was used to calculate the effective binding constants (K_b_) [45].

$$\frac{A_{0}}{A-A_{0}}=\frac{{Ԑ}_{G}}{{Ԑ}_{H\_G}-{Ԑ}_{G}}+\frac{{Ԑ}_{G}}{{Ԑ}_{H\_G}-{Ԑ}_{G}}\times \frac{1}{K\left[\mathrm{DNA}\right]}$$

where K is the association/binding constant; A_0_ and A are the absorbances of the drug and its complex with DNA, respectively; and ${Ԑ}_{G}$ and ${Ԑ}_{H\_G}$ are the absorption coefficient of the drug and the drug–DNA complex, respectively. The intercept to slope ratios from the graphic representation of $A_{0}$/($A-A_{0}$) vs. 1/[DNA] yielded the association constant. Graphs depicting [DNA] vs. [DNA]/${Ԑ}_{a}-{Ԑ}_{f}$, where ${Ԑ}_{a}$ (or ${Ԑ}_{G}$) represents the absorption coefficient of the drug and ${Ԑ}_{f}$ (or ${Ԑ}_{H\_G}$) is the absorption coefficient of the drug–DNA complex.

#### 3.8.2. In Vitro Antitumor Activity

HCT116 colon cancer cell line was purchased from the Egyptian Holding Company for Biological Products & Vaccines (VACSERA), Giza, Egypt, and then maintained in the tissue culture unit (Pharmacy College Taif University, Taif, Saudi Arabia). The cells were cultured in RPMI-1640 medium, supplemented with 10% heat-inactivated FBS, 50 units/mL of penicillin, and 50 mg/mL of streptomycin, and maintained at 37° in a humidified atmosphere containing 5% CO_2_. The cells were maintained as a “monolayer culture” by serial subculturing. Cell culture reagents were obtained from Lonza (Basel, Switzerland). Cytotoxicity was determined using the SRB method as previously described by Skehan et al. Exponentially growing cells were collected using 0.25% Trypsin–EDTA and seeded in 96-well plates at 1000–2000 cells/well in RPMI-1640-supplemented medium. After 24 h, cells were incubated for 72 h with various concentrations of the tested compounds. Following 72 h treatment, the cells were fixed with 10% trichloroacetic acid for 1h at 4 ºC. Next, wells were stained for 10 min at room temperature with 0.4% SRB (sulforhodamine B) dissolved in 1% acetic acid. Finally, the plates were air-dried for 24 h, and the dye was solubilized with Tris-HCl for 5 min on a shaker at 1600 rpm. Each well’s optical density (OD) was measured spectrophotometrically at 564 nm with an ELISA microplate reader (ChroMate-4300, FL, USA). The IC_50_ values were calculated according to the Boltzmann sigmoidal concentration–response curve equation using the nonlinear regression fitting models. The cytotoxic activity of the investigated compound was tested against a normal cell line aiming to get evidence about the selectivity of the compound.

## 4. Conclusions

In this study, four oxidovanadium(IV) complexes have been synthesized and characterized by several spectroscopic methods. The IR spectra suggested that the ligands have bidentate coordination mode to the vanadium ion. In addition, the molar conductance, EPR, magnetic moment values, and electronic data support a square pyramidal structure for all complexes.

Computational studies were applied to demonstrate the optimization geometry and essential quantum parameters and confirm their biological efficiency. The metal complexes showed a square-pyramidal geometry arrangement around the metal ion which was agreeable with experimental results. The form coordination bonds length presented strength bonded between oxidovanadium(IV) and the investigated ligand to form stable complexes. The bioactivity study was varied and involved DNA-binding, molecular docking, QSAR, and cytotoxicity analyses. DNA binding results revealed two behaviors for the synthesized oxidovanadium(IV) complexes with an increase in CT-DNA concentration, hyperchromic with electrostatic or grooves binding modes and hypochromic signifying an intercalation binding mode. Molecular docking results showed that the [VO(CTZ)_2_] 2H_2_O complex exhibited significant interaction with colon cancer (3IG7) Protein with good selectivity.

QSAR study has given significant information on biological activity by using the MLR method. QSAR model showed a good correlation between the predicted and the experimentally observed inhibitory activities. Based on the molecular docking and QSAR results, [VO(CTZ)_2_] 2H_2_O was selected and tested for its inhibitory activity against colon cancer cell line (HCT116). The selected complex showed higher anticancer activity than the standard cisplatin chemotherapy drug.

## Figures and Tables

**Table 1:** The effective analytical and physical data of ligands and their oxidovanadium (IV) complexes.

Complex	M.wt. (g mol^−1^)	Color	M.P. (°C)	% Found (calc.)			Λm (Ω^−1^ mol^−1^ cm^2^)
				C	H	N	
CBZ	185.2	White	113	45.15	5.41	15.04	1
[VO(SO_4_)(CBZ)] 8H_2_O	493.3	Green	125	17.04 (17.25)	5.31 (5.32)	5.68 (5.60)	7.47
CTZ	461.8	White	140	54.62	5.89	6.07	5.23
[VO(CTZ)_2_] 2H_2_O	878.7	Heavy blue	134	57.41 (57.43)	5.96 (5.97)	6.38 (6.41)	8.85
LOR	371.8	Yellow	225	42.00	2.71	11.30	4.00
[VO(LOR)_2_] SO_4_	906.6	Brown	124	35.71 (35.73)	2.31 (2.30)	9.61 (9.54)	68.85
SCZ	250.1	White	130	42.19	3.19	19.68	1.3
[VO(SO_4_)(SCZ)] 7H_2_O	573.8	Green	209	20.93 (30.10)	4.04 (4.13)	9.76 (9.98)	17.50

**Table 2:** The main bands in IR spectra in the range 4000–500 cm^−1^ for ligands and their oxidovanadium(IV) complexes.

Compound	ν_(C=O)_	ν_(C=N)_	ν_(C=S)_	ν_(S=O)_	ν_(V-O)_	ν_(V-N)_	ν_(V-S)_	ν_asym_, ν_sym_ (SO_4_)
CBZ	1677	-	1271	-	-	-	-	-
VO(SO_4_)(CBZ)] 8H_2_O	1677	-	1279	-	596	422	-	1310–1035
CTZ	1739	-	-	-	-	-	-	-
[VO(CTZ)_2_] 2H_2_O	1599, 1409	-	-	-	544			-
LOR	1647	1591	-	-	-	-	-	
[VO(LOR)_2_] SO_4_	1668	1621	-	-	521	496		
SCZ	-	1670	-	1271	-	-	-	
[VO(SO_4_)(SCZ)] 7H_2_O	-	1671	-	1033	589	-	424	1280–1011

**Table 3:** Electronic spectra data of ligand and their oxidovanadium(IV) complexes and their magnetic moment values.

Compound	π–π*	n–π*	µeff. (B.M.)	d-d (broad)
CBZ	266	-		-
[VO(SO_4_)(CBZ)] 8H_2_O	273	-	1.74	850
CTZ	262	280		-
[VO(CTZ)_2_] 2H_2_O	260	331	1.75	900
LOR	274	394		-
[VO(LOR)_2_] SO_4_	263	311	1.76	850
SCZ	262	275		-
[VO(SO_4_)(SCZ)] 7H_2_O	282	373	1.75	870

**Table 4:** EPR parameter in DMF at room temperature of oxidovanadium(IV) complexes.

Complex	g	A
[VO(SO_4_)(CBZ)] 8H_2_O	1.992	96
[VO(CTZ)_2_] 2H_2_O	1.980	106
[VO(LOR)_2_] SO_4_	1.982	105
[VO(SO_4_)(SCZ)] 7H_2_O	1.984	108

**Table 5:** The optimized geometry and V=O bond length of the metal complexes in the gas phase using DFT method LANL2DZ basis sets.

Compound	Optimized Geometry
[VO(SO_4_)(CBZ)] 8H_2_O	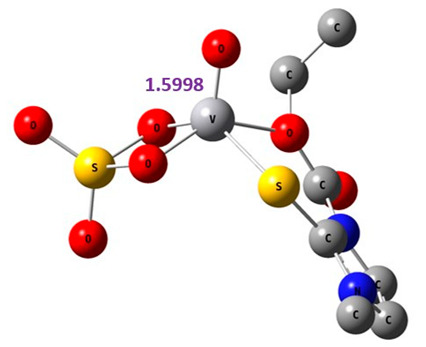
[VO(CTZ)_2_] 2H_2_O	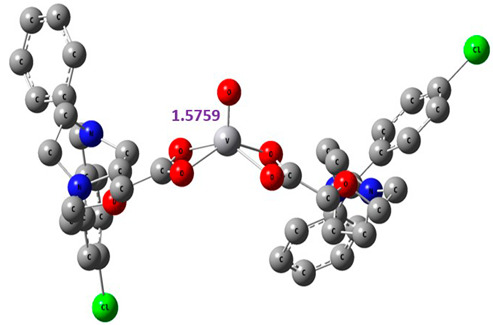
[VO(LOR)_2_]SO_4_	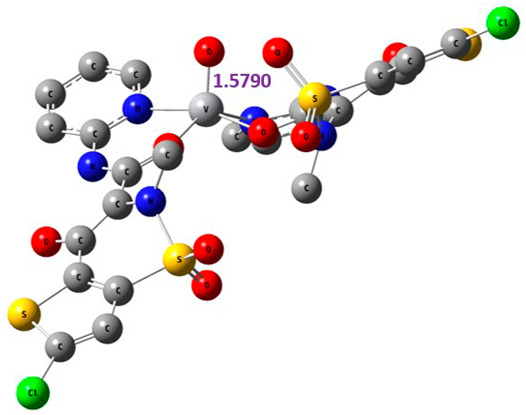
[VO(SO_4_)(SCZ)] 7H_2_O	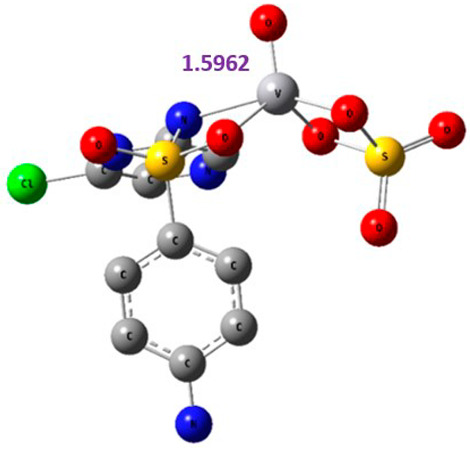

**Table 6:** Bond lengths and stretching frequencies for V=O Bonds in synthesized oxidovanadium(IV) complexes, experimental, and theoretical values.

Complex	ν(V=O)		V=O Radius	
	Exp	DFT	Exp	DFT
[VO(SO_4_)(CBZ)] 8H_2_O	980	1037	1.607	1.599
[VO(CTZ)_2_] 2H_2_O	963	1068	1.616	1.579
[VO(LOR)_2_]SO_4_	968	1075	1.613	1.576
[VO(SO_4_)(SCZ)] 7H_2_O	987	1047	1.603	1.596

**Table 7:** E_HOMO_, E_LUMO_, Eg, and the ligand’s quantum parameters and complexes using B3LYP/LANL2DZ.

Compound	HUMO	LUMO	∆E	x	η	σ	Pi	σ	S	ω	ΔN Max
CBZ	−5.55	−1.00	4.55	3.28	2.28	0.44	−3.28	0.44	1.14	2.36	1.44
VO-CBZ	−7.09	−3.27	3.83	5.18	1.91	0.52	−5.18	0.96	0.96	2.59	2.71
SCZ	−6.75	−2.78	3.97	4.77	1.99	0.50	−4.77	0.99	0.99	2.38	2.40
VO-SCZ	−7.41	−3.64	3.77	5.53	1.89	0.53	−5.53	0.94	0.94	2.76	2.93
LOR	−3.96	−1.99	1.97	2.98	0.98	1.02	−2.98	0.49	0.49	1.49	3.03
VO-LOR	−3.93	−2.74	1.19	3.34	0.59	1.68	−3.34	0.30	0.30	1.67	5.62
CTZ	−6.59	−4.68	1.91	5.64	0.95	1.05	−5.64	0.48	0.48	2.82	5.91
VO-CTZ	−10.26	−8.84	1.42	10.05	1.21	0.83	−10.05	0.61	0.61	5.03	8.31

**Table 8:** The spectral parameters for the interaction of oxidovanadium(IV) complexes and their ligands with DNA.

Compound	K_b_ (M^−1^)	λ_max_ Free (nm)	λ_max_ Bound (nm)	Type of Chromism
CBZ	8.33 × 10^5^	261	255	Hyperchromic
[VO(SO_4_)(CBZ)] 8H_2_O	5.00 × 10^5^	265	256	Hyperchromic
CTZ	1.00 × 10^6^	263	263	Hyperchromic
[VO(CTZ)_2_] 2H_2_O	1.40 × 10^6^	265	259	Hyperchromic
LOR	8.33 × 10^5^	395	376	Hypochromic
[VO(LOR)_2_] SO_4_	1.20 × 10^6^	281	274	Hyperchromic
SCZ	1.00 × 10^6^	265	257	Hyperchromic
[VO(SO_4_)(SCZ)] 7H_2_O	1.25 × 10^6^	262	256	Hyperchromic

**Table 9:** Docking score and energies of ligands and their oxidovanadium(IV) complexes with 3IG7 protein.

Compound	S	rmsd_Refine	E_conf	E_Place	E_Refine
CBZ	−5.13	0.95	−16.39	−50.34	−27.37
[VO(SO_4_)(CBZ)] 8H_2_O	−5.95	2.88	−624.55	−49.81	−15.08
CTZ	−7.60	1.41	129.88	−92.44	−28.38
[VO(CTZ)_2_] 2H_2_O	−9.81	1.86	−658.30	−79.50	−29.23
LOR	−6.58	1.04	2.82	−87.23	−36.53
[VO(LOR)_2_] SO_4_	−6.83	2.35	−358.53	−1.13	−37.58
SCZ	−5.83	1.93	−40.53	−57.04	−31.67
[VO(SO_4_)(SCZ)] 7H_2_O	−6.90	1.16	−797.18	−79.19	−9.94

**Table 10:** 2D and 3D Docking interaction of the oxidovanadium(IV) complexes with colon cancer protein (PDB code = 3IG7).

Compound	2D Snapshot	3D Snapshot
CBZ	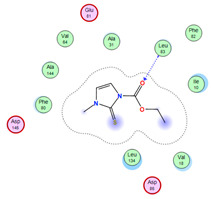	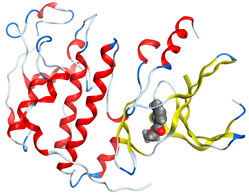
[VO(SO_4_)(CBZ)] 8H_2_O	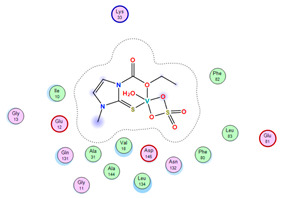	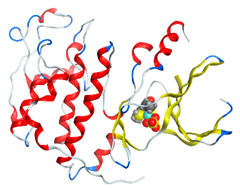
CTZ	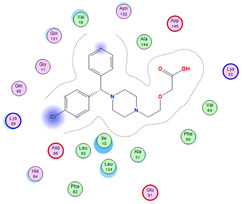	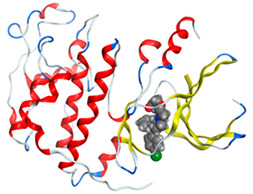
[VO(CTZ)_2_] 2H_2_O	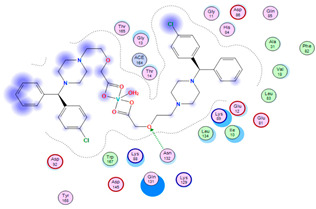	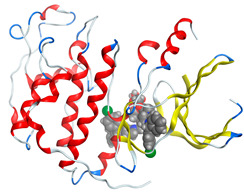
LOR	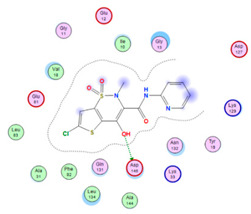	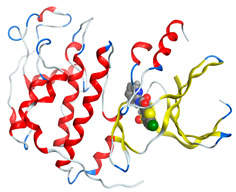
[VO(LOR)_2_]SO_4_	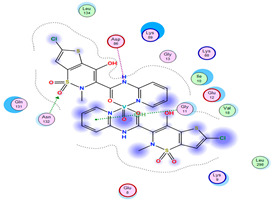	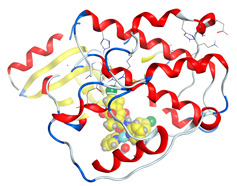
SCZ	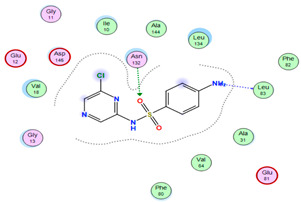	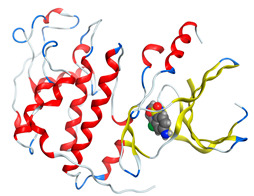
[VO(SO_4_)(SCZ)] 7H_2_O	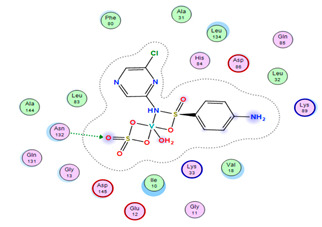	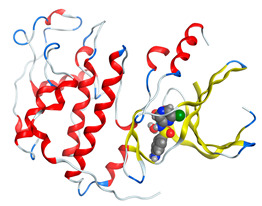
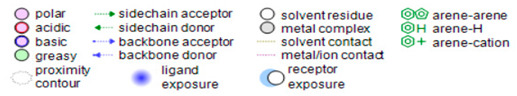		

**Table 11:** The predicted IC_50_ of all synthesized oxidovanadium(IV) complexes and their free ligands (testing set) against HCT116 cell.

Compound	Predicted IC_50_ (µM)
CBZ	42.43
[VO(SO_4_)(CBZ)] 8H_2_O	39.87
CTZ	12.30
[VO(CTZ)_2_] 2H_2_O	1.45
LOR	10.26
[VO(LOR)_2_] SO_4_	2.61
SCZ	17.62
[VO(SO_4_)(SCZ)] H_2_O	37.06

**Table 12:** Anticancer activity of [VO(CTZ)_2_] 2H_2_O compared to Cisplatin and the stander division value.

Complex	IC_50_ ± SD (µM)
[VO(CTZ)_2_] 2H_2_O	2.11 ± 0.02
Cisplatin	2.13 0.03

## Data Availability

All relevant data are within the manuscript and its Supporting information.

## Supplementary Materials

Table S1: The experimental data of oxidovanadium(IV) complexes, Table S2: Thermogravimetric analysis data for the oxidovanadium(IV) complexes, Table S3: Kinetic parameters of thermal decomposition steps for oxidovanadium(IV) complexes, Table S4: Coats-Redfern (CR) and Horowitz–Metzger (HM) of the oxidovanadium(IV) complexes, Table S5: HOMO and LUMO plots of the free ligands and their metal complexes using DFT/B3LY, Table S6: Docking interaction calculation of all oxidovanadium(IV) complexes with colon cancer protein (3IG7), Table S7: The experimental and the predicted IC50 values of some of the training set against HCT116 cell, Table S8: Docking score and energies of Co-crystalline compound (positive control) and four amino acids (negative control), Table S9: 2D and 3D Docking interaction of the Co-crystalline compound (positive control) and four amino acids (negative control), Figure S1: Molar ratio method plots of oxidovanadium(IV) complexes, Figure S2: Some of the structures of imidazole and oxidovanadium(IV) compounds (training) that were used in the QSAR with their activity against HCT 116, Figure S3:The FT-IR spectra of free ligands and their oxidovanadium(IV) complex, Figure S4: UV-vis Spectra of free ligands and their oxidovanadium(IV) complexes (inset shows the d-d transition the metal complex), Figure S5: EPR spectra of oxidovanadium(IV) complexes in DMF and in room temperature, Figure S6: The TGA curves of synthesized oxidovanadium(IV) complex, Figure S7: The optimized geometry of the free ligands gas phase using DFT method LANL2DZ basis sets, Figure S8: Absorption spectra of free ligands and their complex in the presence of increasing DNA concentration.

## Sample Availability

Samples of the compounds available from the authors.
