# Supplementary material for: QSAR Modeling, Molecular Docking and Cytotoxic Evaluation for Novel Oxidovanadium(IV) Complexes as Colon Anticancer Agents
Source: Molecules. 2022 Jan 19;27(3):649. doi: 10.3390/molecules27030649 (PMC8838224; doi:10.3390/molecules27030649)
Supplement: Supplementary file 1 [file molecules-27-00649-s001.zip › molecules-1521738-supplementary.pdf]

QSAR Modeling, Molecular Docking and Cytotoxic Evaluation for Novel Oxidovanadium(IV) Complexes as Colon Anticancer Agents

Fatimah Y. Alomari [a], Abeer A. Sharfalddin[b], Magda H Abdellattif[c], Doaa Domyati[d], Amal S. Basaleh[b], Mostafa A. Hussien [b,e*]

[a] Chemistry Department, College of Science, Imam Abdulrahman Bin Faisal University, P.O. Box 76971, Dammam 31441, Saudi Arabia.

[b] Department of Chemistry, Faculty of Science, King Abdulaziz University, P.O. Box 80203 Jeddah 21589, Saudi Arabia.

[c] Deanship of Scientific Research, Pharmacy College, Taif University, P.O. Box 888 Taif 21974, Saudi Arabia.

[d] Department of Chemistry, College of Science, University of Jeddah, P.O. BOX 80327, Jeddah 21589, Saudi Arabia

[e]Department of Chemistry, Faculty of Science, Port Said University, Port Said 42521, Egypt

Corresponding author:

Mostafa A. Hussien*

maabdulaal@kau.edu.sa

Table S1 The experimental data of oxidovanadium(IV) complexes.

NO.	Metal conc. (×10^{-4} moles)	Ligand conc. (×10^{-4} moles)	[M]/([M] + [L])	$[VO(SO_4)(CBZ)] 8H_2O$	$[VO(CTZ)_2] 2H_2O$	$[VO(LOR)_2] SO_4$	$[VO(SO_4)(SCZ)] 7H_2O$
					Absorbance		
				at 256 nm	at 208 nm	at 389 nm	at 210 nm
**1**	0.72	2.52	0.22	1.15	1.12	0.75	0.86
**2**	0.72	2.16	0.25	1.06	1.03	0.62	0.83
**3**	0.72	1.80	0.28	0.95	0.93	0.52	0.76
**4**	0.72	1.44	0.33	0.85	0.84	0.42	0.69
**5**	0.72	1.08	0.40	0.72	0.71	0.31	0.57

**6**	0.72	0.72	0.50	0.61	0.67	0.22	0.52
**7**	0.72	0.36	0.60	0.50	0.59	0.11	0.46
**8**	0.72	0.00	1.00	0.38	0.49	0.07	0.41

Table S2 Thermogravimetric analysis data for the oxidovanadium(IV) complexes.

Complex	Step	Temp. range (°C)	Weight loss % found (calc)	Assignments	Total mass loss/% found (calc)	Final solid-state residue %
$[VO(SO_4)(CBZ)]$ $8H_2O$	1st	25–218	55.19 (55.15)	$7H_2O$+ $C_4H_7N_2O_3S$	82.74 (82.54)	VO_2 17.63 (17.45)
	2nd	218–800	27.55 (27.39)	$C_5H_3O_2S$		
$[VO(CTZ)_2]$ $2H_2O$	1st	55–100	4.09 (4.12)	$2H_2O$	90.55 (90.45)	VO_2 9.44 (9.55)
	2nd	100–788	86.46 (86.33)	$C_{42}H_{48}Cl_2$ N_4O_5		
$[VO(LOR)_2]$ SO_4	1st	119–341	42.36 (42.35)	$C_7H_6Cl_2O_{10}S_2$	90.62 (90.85)	VO_2 9.38 (9.15)
	2nd	341–552	48.26 (48.09)	$C_{19}H_{12}N_6OS_3$		
$[VO(SO_4)(SCZ)]$ $7H_2O$	1st	25–232	69.37 (70.01)	$7H_2O$+$C_6H_8N_2O_4S_2$	85.59 (86.54)	VO_2 14.40 (13.45)
	2nd	232–998	16.22 (16.53)	C_4HN_2O		

Table S3 Kinetic parameters of thermal decomposition steps for oxidovanadium(IV) complexes

**Complex**	**Temp. range C°**	**Method**	**Ea (kJmol^{-1})**	**A (s^{-1})**	**ΔS (J mol^{-1} K^{-1})**	**ΔH (kJmol^{-1})**	**ΔG (kJ mol^{-1})**	**Correlation coefficient (R)**
$[VO(SO_4)(CBZ)]$ $8H_2O$	25-218	CR	4.88×10^4	2.03×10^3	-1.85×10^2	4.52×10^4	1.25×10^5	0.99
		HZ	5.62×10^4	3.58×10^4	-1.61×10^2	5.26×10^4	1.22×10^5	0.99
		Average	5.25×10^4	1.90×10^4	-1.73×10^2	4.89×10^4	1.24×10^5	0.99
	218-800	CR	4.37×10^4	3.12	-2.42×10^2	3.85×10^4	1.89×10^5	0.98
		HZ	5.43×10^4	9.95×10	-2.13×10^2	4.91×10^4	1.82×10^5	0.99
		Average	4.90×10^4	5.13×10	-4.91×10^2	4.38×10^4	1.85×10^5	0.98
$[VO(CTZ)_2]$ $2H_2O$	25-100	CR	1.20×10^5	1.91×10^3	-1.83×10^2	1.17×10^5	1.87×10^5	0.99
		HZ	1.44×10^4	4.77×10	-2.52×10^2	1.17×10^4	9.56×10^4	0.99
		Average	6.71×10^4	9.55×10^2	-2.18×10^2	6.43×10^5	1.37×10^5	0.99
$[VO(LOR)_2]$ SO_4	119-341	CR	4.51×10^4	5.88×10^2	-1.96×10^2	4.11×10^4	1.34×10^5	0.99

		HZ	3.36×10^{4}	1.45×10	-2.27×10^{2}	2.97×10^{4}	1.38×10^{5}	0.98
		Average	3.93×10^{4}	3.01×10^{2}	-2.11×10^{2}	2.54×10^{4}	1.36×10^{5}	0.98
	341-552	CR	5.95×10^{4}	1.49×10	-2.30×10^{2}	5.34×10^{4}	2.22×10^{5}	0.99
		HZ	1.02×10^{5}	7.42×10^{4}	-1.59×10^{2}	9.62×10^{4}	2.13×10^{5}	0.99
		Average	8.09×10^{4}	3.71×10^{4}	-1.95×10^{2}	7.48×10^{4}	2.17×10^{5}	0.99
$[VO(SO_4)(SCZ)]7H_2O$	25–232	CR	4.93×10^{4}	4.57×10^{2}	-1.97×10^{2}	4.57×10^{4}	1.32×10^{5}	0.99
		HZ	5.69×10^{4}	3.98×10^{4}	-1.60×10^{2}	5.33×10^{4}	1.23×10^{5}	0.99
		Average	5.31×10^{4}	2.02×10^{4}	-1.79×10^{2}	4.95×10^{4}	1.27×10^{5}	0.99
	232-998	CR	3.56×10^{4}	7.15×10	-2.37×10^{2}	2.86×10^{4}	2.28×10^{5}	0.99
		HZ	9.92×10^{4}	4.24×10^{3}	-1.84×10^{2}	9.22×10^{4}	2.47×10^{5}	0.99
		Average	6.74×10^{4}	2.12×10^{3}	-2.11×10^{2}	6.04×10^{4}	2.37×10^{5}	0.99

Table S4 Coats-Redfern (CR) and Horowitz–Metzger (HM) of the oxidovanadium(IV) complexes.

Complex	Stage	Horowitz–Metzger (HM)	Coats-Redfern (CR)
$[VO(SO_4)(CBZ)]\ 8H_2O$	25-218	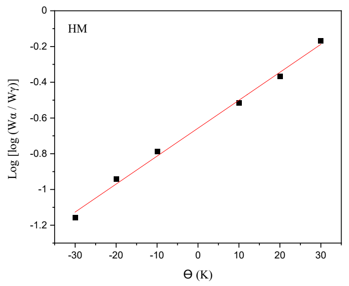	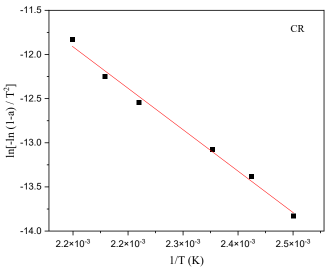
	218-800	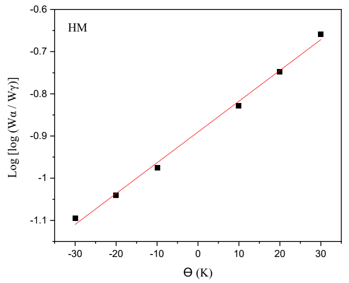	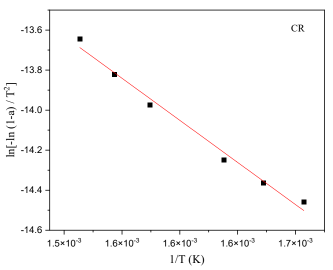
$[VO(CTZ)_2]\ 2H_2O$	25-100	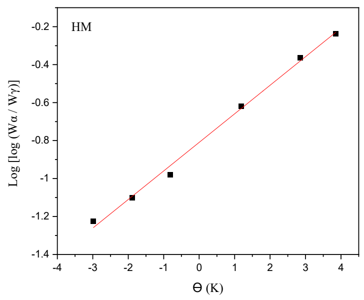	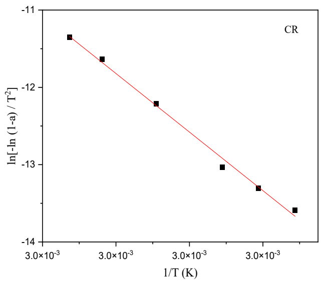

Complex	Stage	Horowitz–Metzger (HM)	Coats-Redfern (CR)
	100 - 788	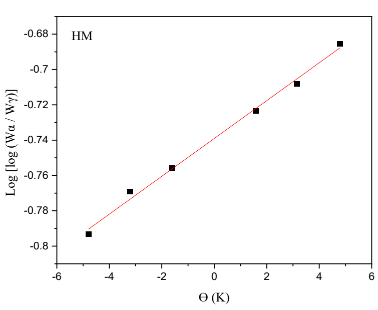	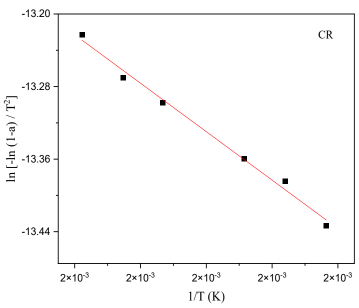

Complex	Stage	Horowitz–Metzger (HM)	Coats-Redfern (CR)
$[VO(LOR)_2]SO_4$	119-341	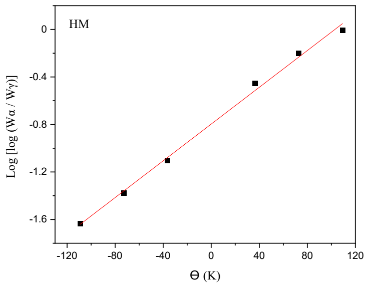	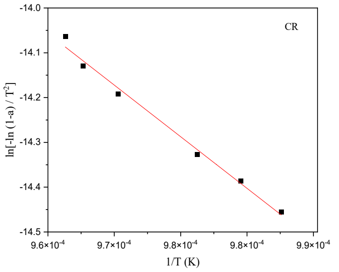
	341-552	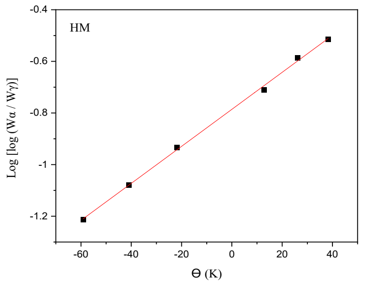	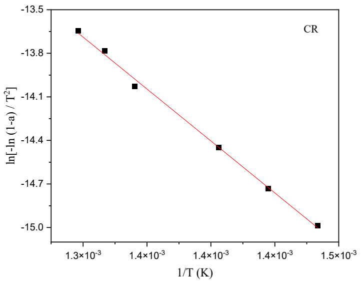
$[VO(SO_4)(SCZ)]7H_2O$	25–232	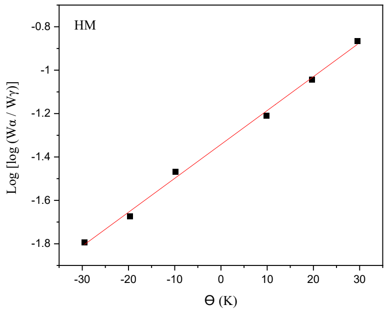	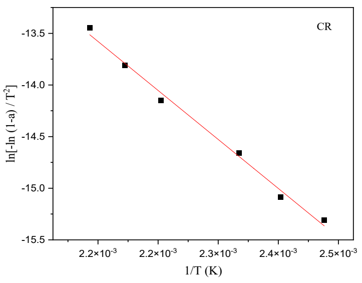
	232-998	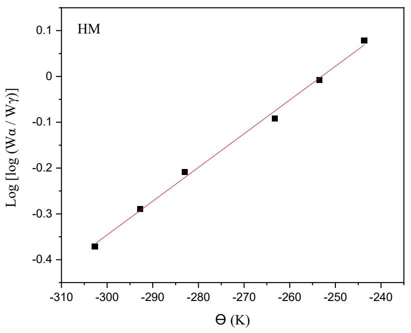	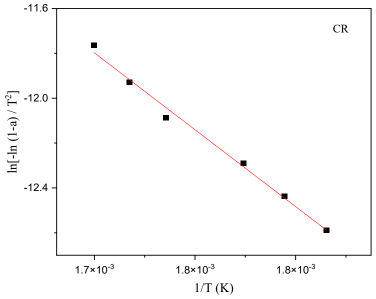

Table S 5 HOMO and LUMO plots of the free ligands and their metal complexes using DFT/B3LY.

Compound	HOMO	LUMO
SCZ	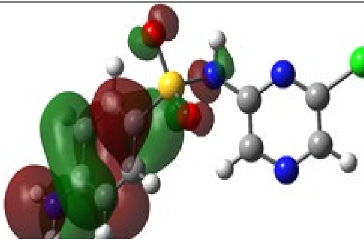	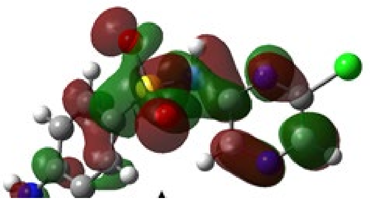
VO-SCZ	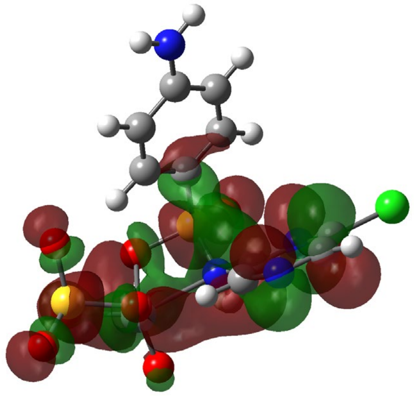	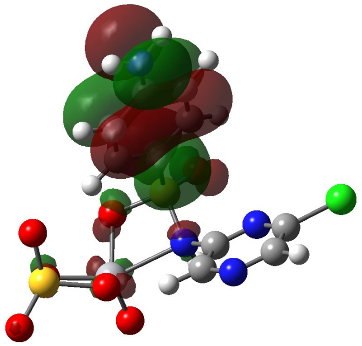
CBZ	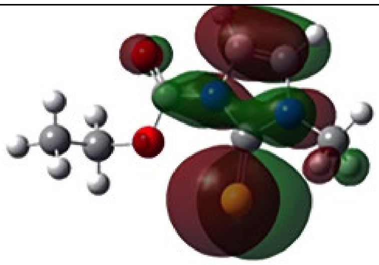	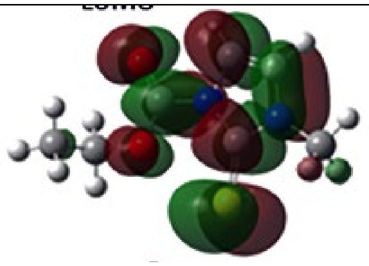
VO-CBZ	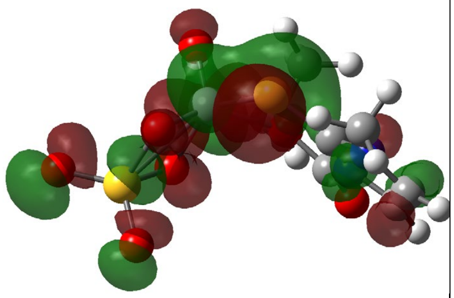	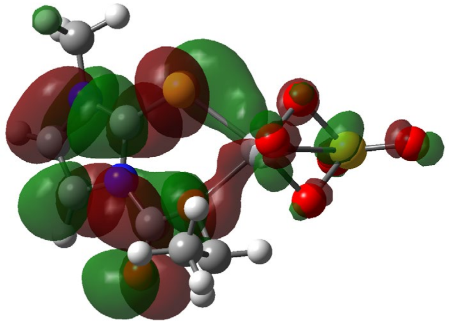

CTZ	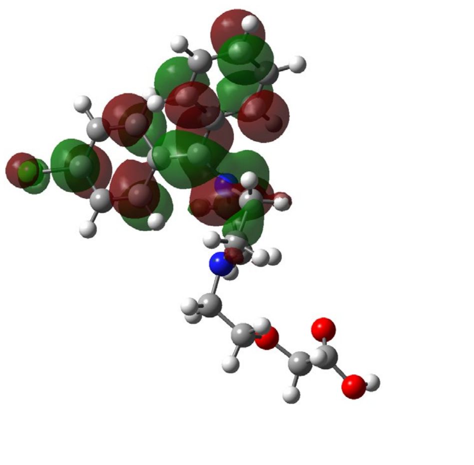	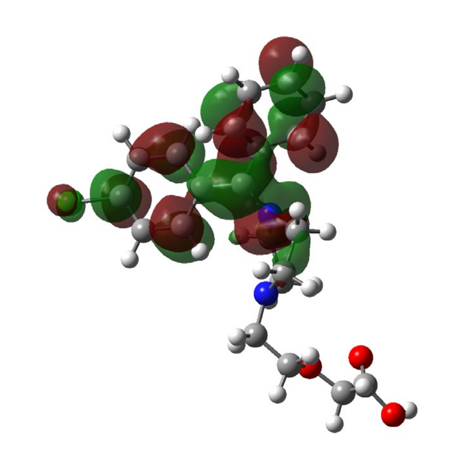
VO-CTZ	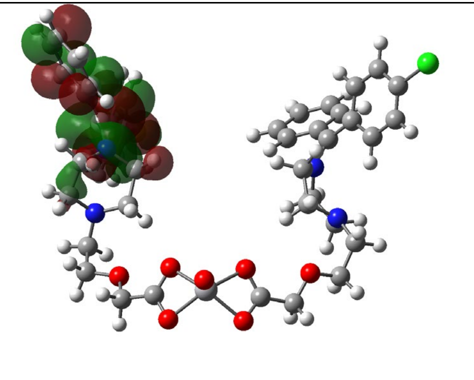	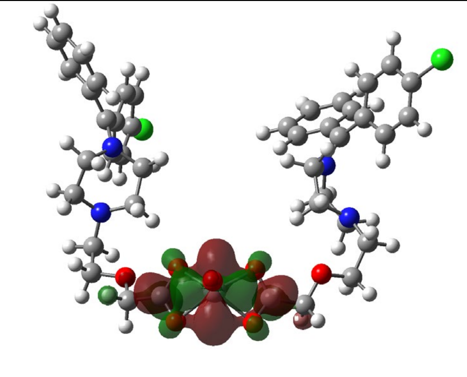
LOR	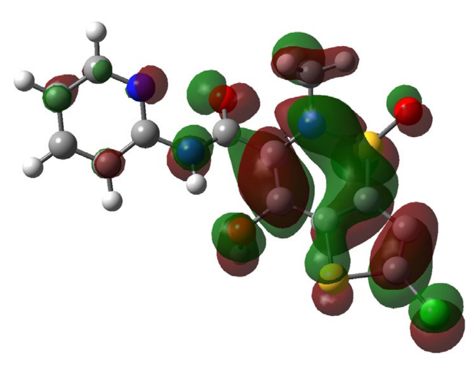	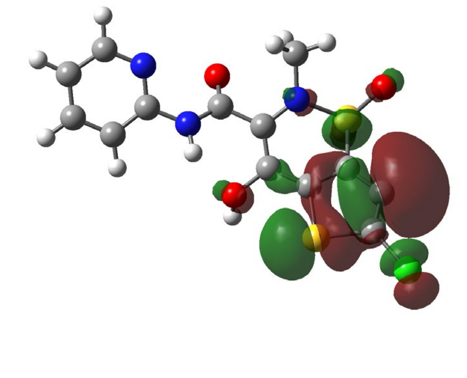
VO- LOR	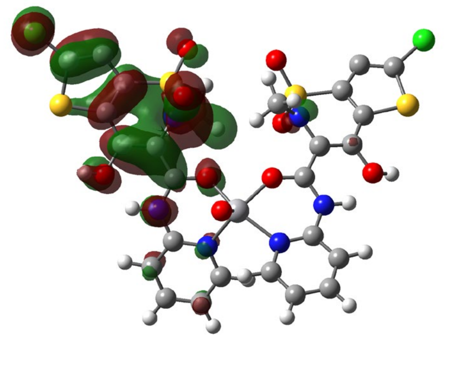	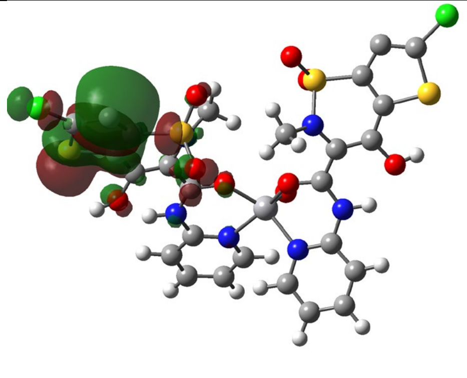

Table S6 Docking interaction calculation of all oxidovanadium(IV) complexes with colon cancer protein (3IG7).

Compound		Receptor	Interaction	Distance E	(kcal/mol)
CBZ	O 22	N LEU 83 (A)	H-acceptor	3.19	-2.3
$[VO(SO_4)(CBZ)]8H_2O$	No measurable interactions				
CTZ	No measurable interactions				
$[VO(CTZ)_2]\ 2H_2O$	O 45	ND2 ASN 132(A)	H-acceptor	3.10	-2.0
LOR	O 21	OD1 ASP 145(A)	H-donor	2.82	-6.6
$[VO(LOR)_2]SO_4$	O 66	ND2 ASN 132(A)	H-acceptor	2.94	-1.8
	N 44	OD1 ASP 86(A)	Ionic	3.92	-0.7
	6-ring	CA GLY 11 (A)	pi-H	4.44	-1.0
SCZ	N 23	N LEU 83 (A)	H-acceptor	3.14	17.9
	O 26	ND2 ASN 132(A)	H-acceptor	3.02	-1.0
$[VO(SO_4)(SCZ)_2]7H_2O$	O 35	ND2 ASN 132(A)	H-acceptor	3.37	-1.2

Table S7 The experimental and the predicted IC_{50} values of some of the training set against HCT116 cell.

Compounds	IC_{50} (experimental)	IC_{50} (predicted)	Residual
1a	0.95 ± 0 .20	1.72	-0.16099
2a	0.34 ± 0.08	4.21	-0.93851
3a	0.05 ± 0.21	3.72	-0.82802
4a	0.31 ± 0.08	1.06	1.393651
5a	29.23 ± 3.28	36.43	-1.43153
6a	32.90 ± 2.97	34.935	-0.43057
7a	24.39 ± 4.28	27.11	-0.69201
8a	28.70 ± 2.30	23.92	1.129149
9a	33.40 ± 3.30	31.06	0.430793
10a	>50.00	41.93	1.794515
11a	>50.00	51.19	-0.26647

Table S8. Docking score and energies of Co-crystalline compound (positive control) and four amino acids (negative control).

compound	S	rmsd_refine	E_conf	E_place	E_score1	E_refine	E_score2
Co-crystalline compound	-7.30	1.47	-115.39	-30.76	-8.60	-26.01	-7.30
	-7.15	1.30	-113.25	-31.40	-8.52	-25.61	-7.15
	-7.11	2.09	-120.47	-32.17	-8.51	-37.24	-7.11
	-6.91	1.30	-108.56	-50.41	-9.53	-32.68	-6.91
	-6.83	1.05	-115.80	-43.13	-9.18	-26.67	-6.83
guanine	-1.37	4.79	-119.69	-32.70	-7.49	-20.31	-1.37
	-1.29	1.85	-119.29	-51.86	-7.56	-15.73	-1.29
	-1.19	2.35	-119.43	-49.12	-7.43	-13.91	-1.19
	-1.08	2.16	-119.54	-35.00	-6.85	-21.40	-1.08
	-1.07	0.90	-119.44	-39.01	-7.27	-13.49	-1.07
adenine	-1.40	0.30	-104.28	-60.33	-6.35	-18.50	-1.40

	-1.11	1.24	-104.20	-35.37	-5.90	-18.79	-1.11
	-1.10	3.26	-104.22	-36.40	-5.96	-15.12	-1.10
	-1.03	2.13	-104.12	-38.24	-5.95	-19.33	-1.03
	-1.02	1.77	-104.31	-30.58	-6.53	-15.87	-1.02
thymine	-1.26	1.27	-129.54	-29.36	-5.67	-20.51	-1.26
	-0.90	1.99	-129.42	-33.59	-5.64	-23.78	-0.90
	-0.84	1.28	-129.53	-13.76	-6.29	-13.57	-0.84
	-0.83	5.19	-128.85	-28.94	-5.83	-16.47	-0.83
	-0.77	1.94	-129.48	-38.45	-5.70	-16.28	-0.77
cytosine	-0.06	1.25	-145.94	-51.77	-5.56	-18.76	-0.06
	-0.95	1.43	-146.16	-16.57	-5.31	-14.63	-0.95
	-0.82	1.20	-146.13	-29.13	-5.81	-16.63	-0.82
	-0.76	1.23	-146.10	-19.65	-5.51	-10.82	-0.76
	-0.76	9.29	-146.06	-10.77	-5.29	-16.03	-0.76

Table S9. 2D and 3D Docking interaction of the Co-crystalline compound (positive control) and four amino acids (negative control).

Co-crystalline compound	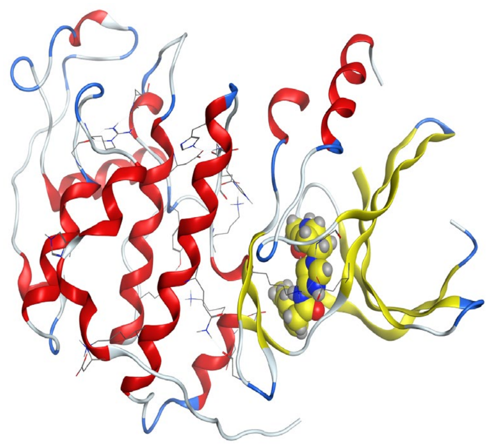	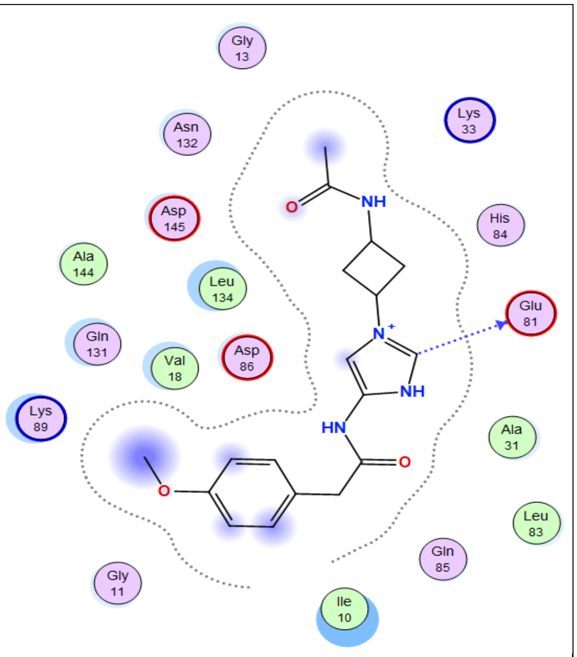

guanine	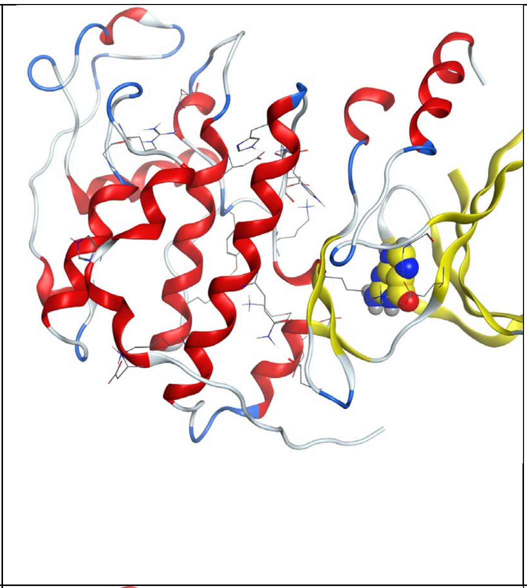	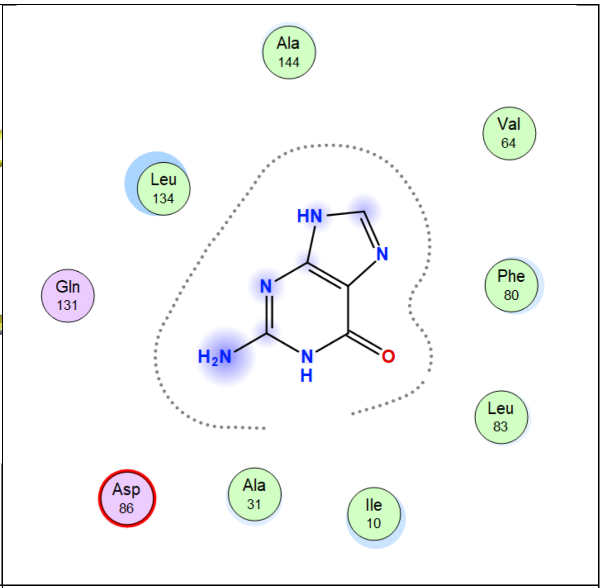
adenine	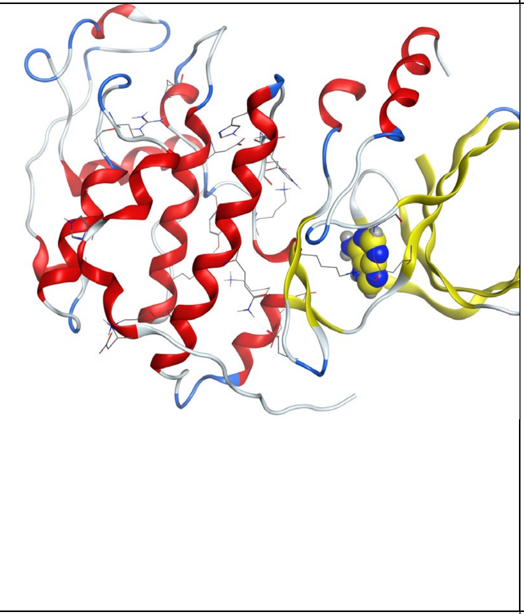	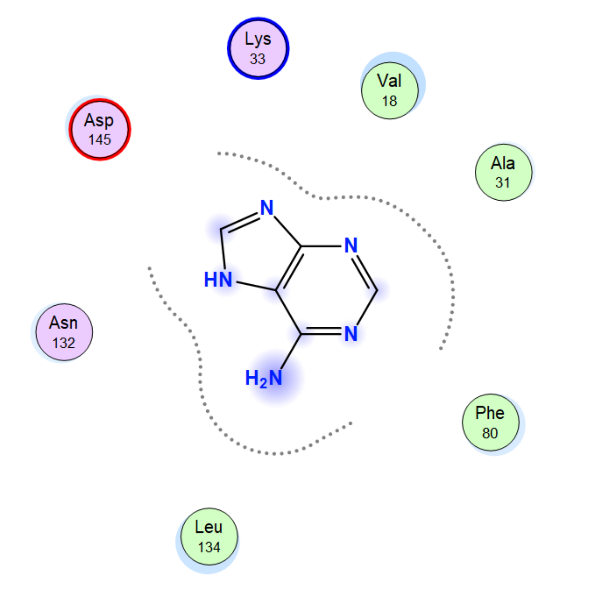
thymine	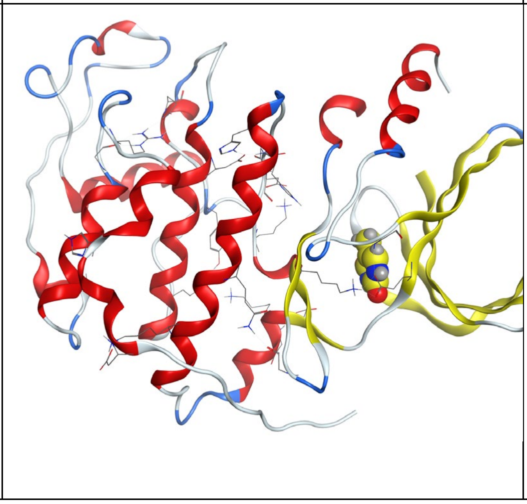	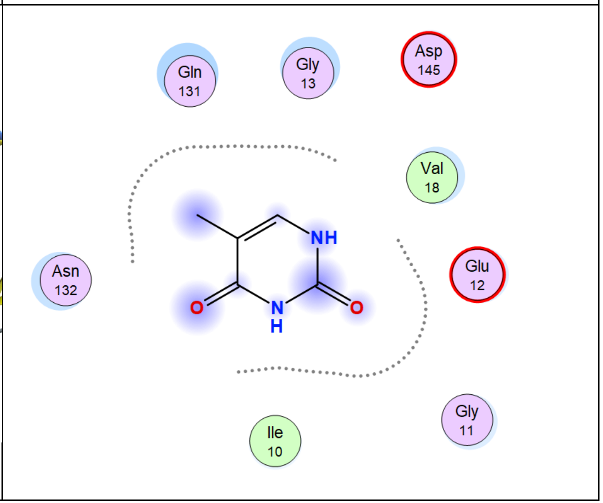

cytosine	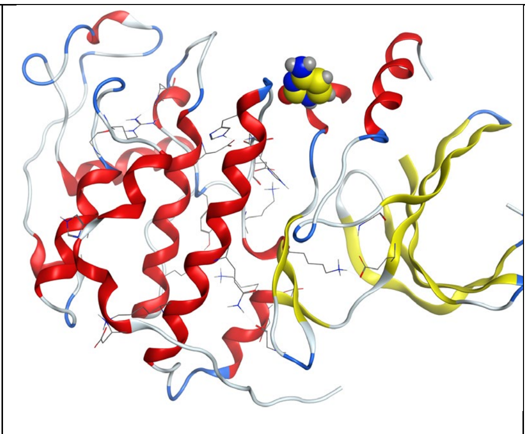	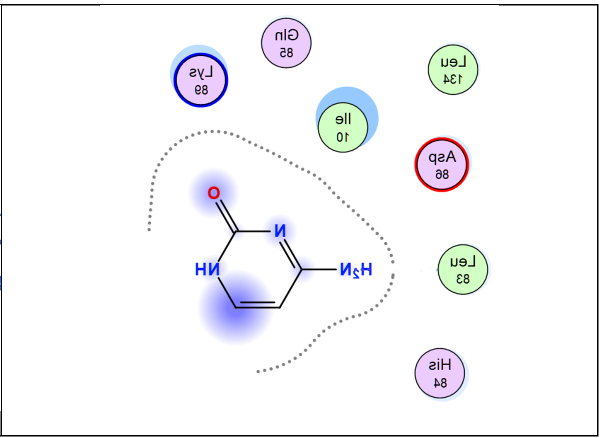

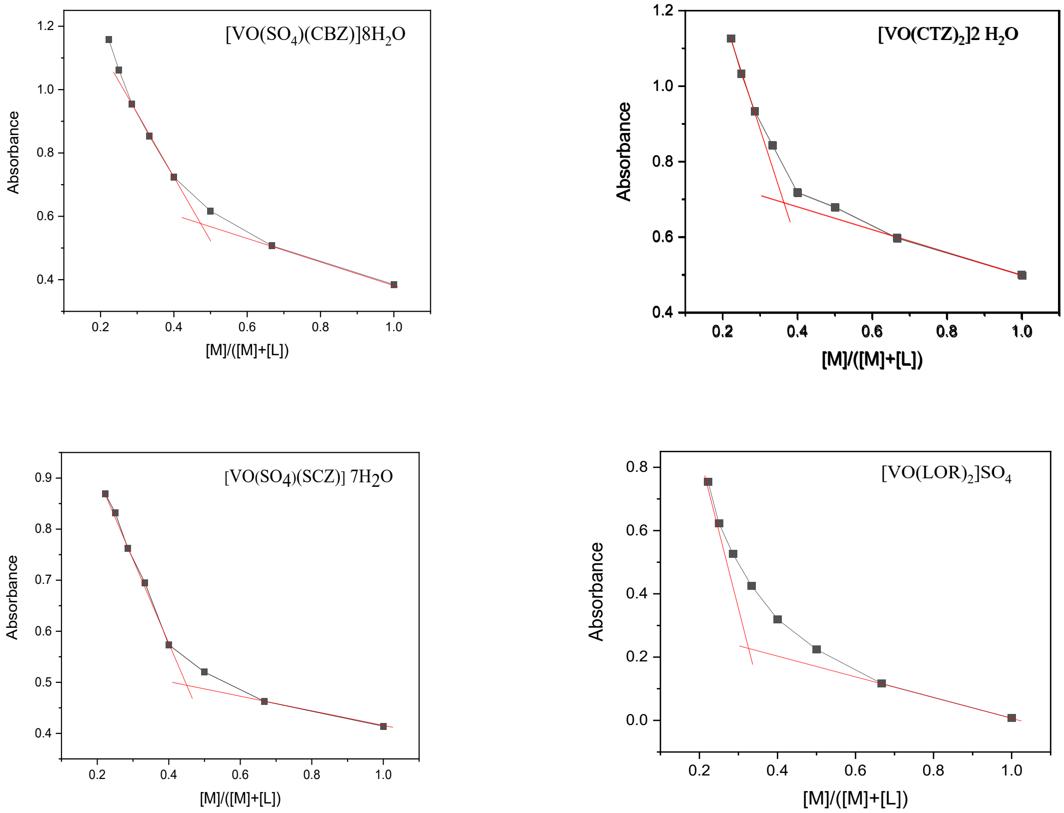

Figure S1 Molar ratio method plots of oxidovanadium(IV) complexes.

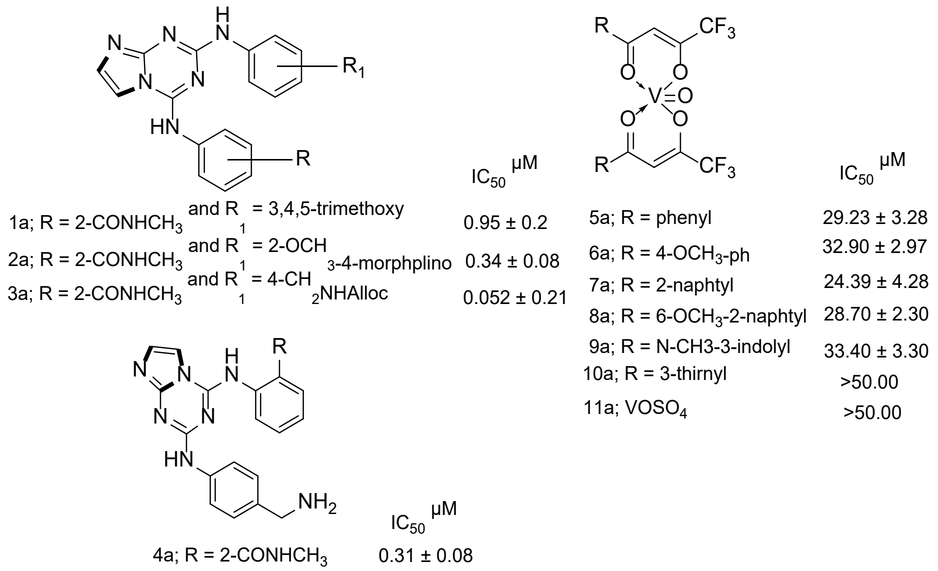

Figure S2. Some of the structures of imidazole and oxidovanadium(IV) compounds (training) that were used in the QSAR with their activity against HCT 116.

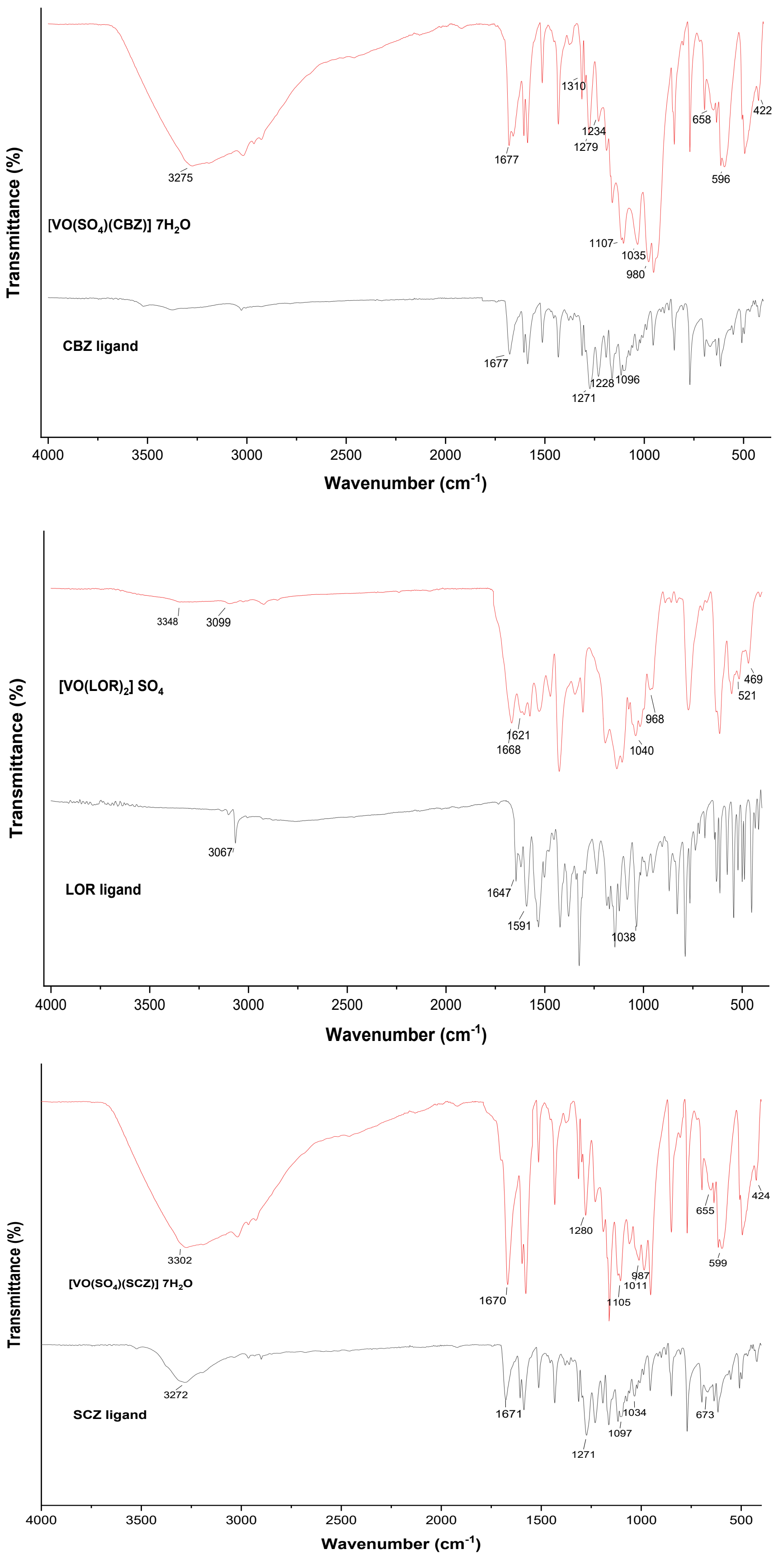

Figure S1 The FT-IR spectra of free ligands and their oxidovanadium(IV*)* complex.

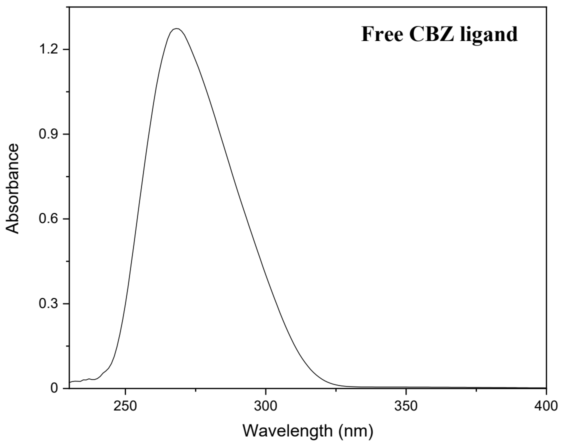
Free CBZ ligand
Absorbance
Wavelength (nm)
0
0.3
0.6
0.9
1.2
250
300
350
400

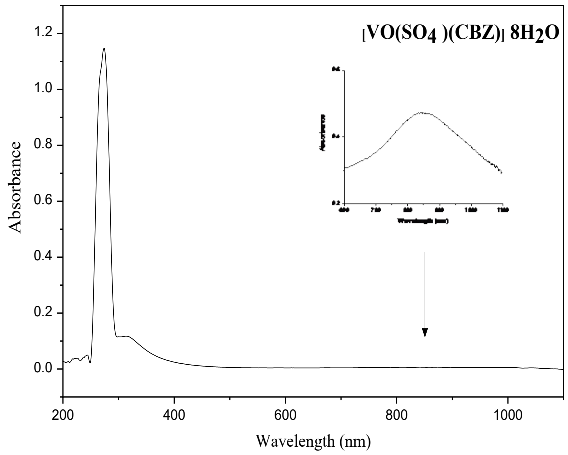
[VO(SO4)(CBZ)] 8H2O
Absorbance
Wavelength (nm)
0.0
0.2
0.4
0.6
0.8
1.0
1.2
200
400
600
800
1000

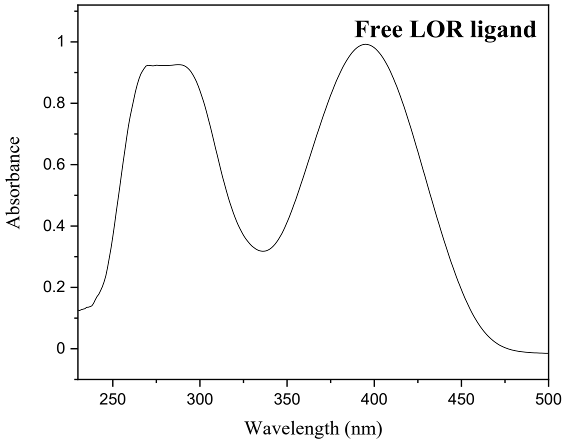
Free LOR ligand
Absorbance
Wavelength (nm)
0
0.2
0.4
0.6
0.8
1
250
300
350
400
450
500

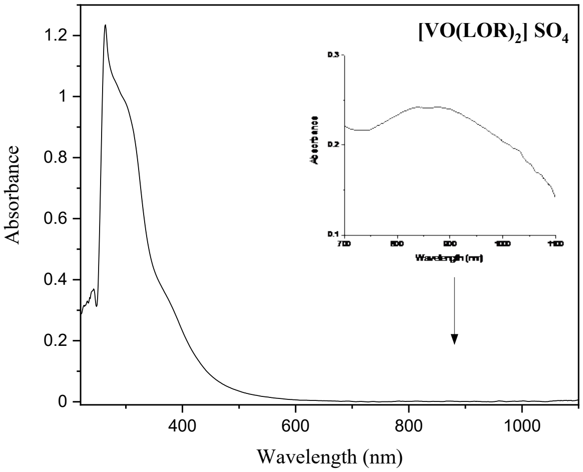
[VO(LOR)2] SO4
Absorbance
Wavelength (nm)
0
0.2
0.4
0.6
0.8
1
1.2
400
600
800
1000

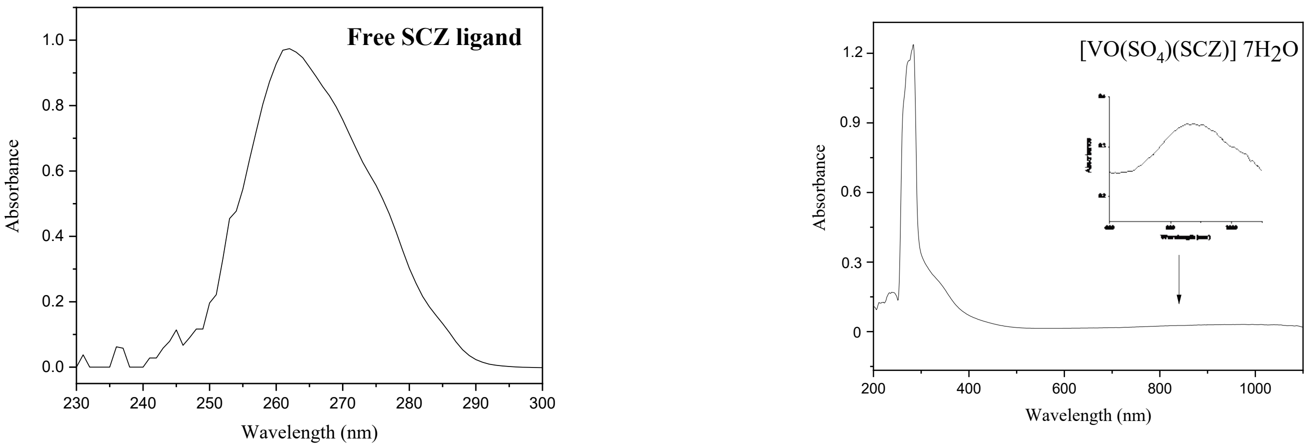

Figure S2 UV-vis Spectra of free ligands and their oxidovanadium(IV) complexes (inset shows the d-d transition the metal complex).

.

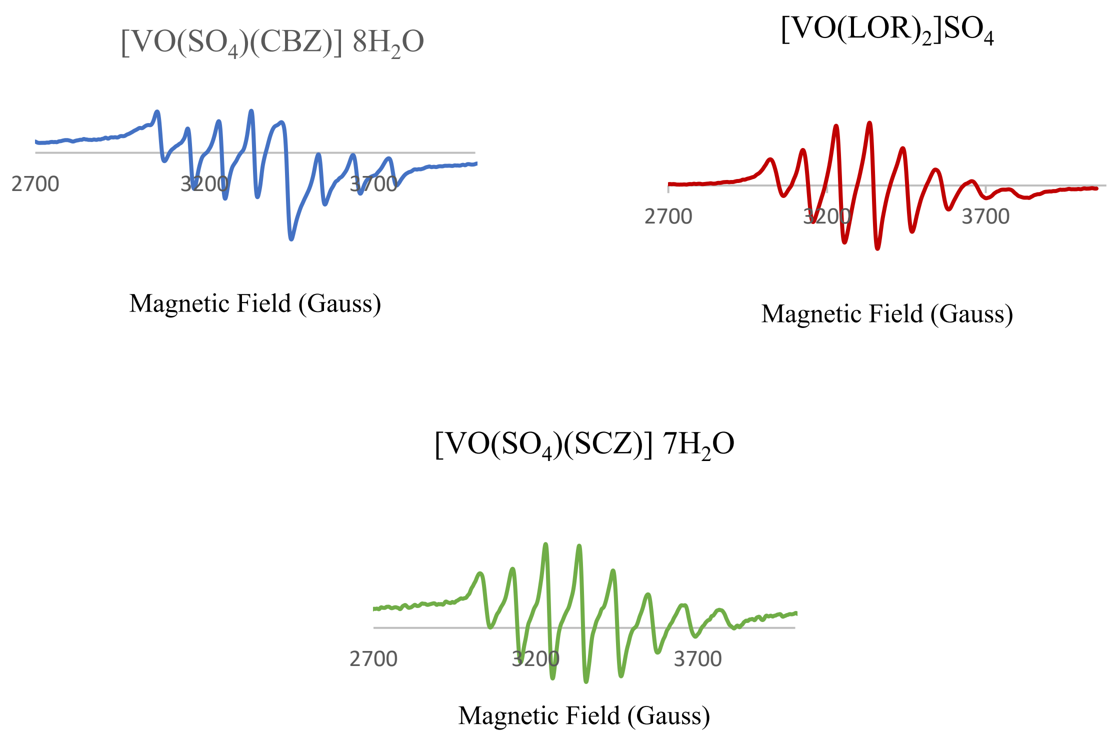

Figure S3 EPR spectra of oxidovanadium(IV*)* complexes in DMF and in room temperature.

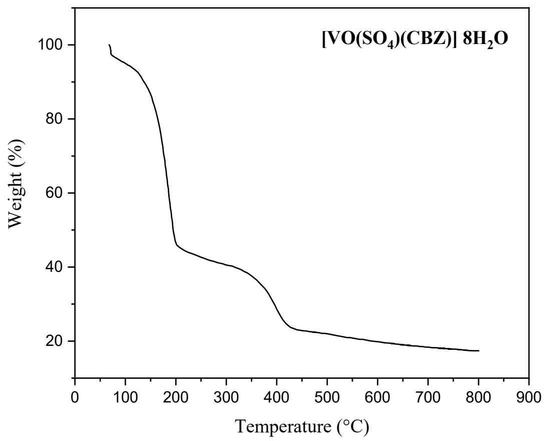
[VO(SO4)(CBZ)] 8H2O
Weight (%)
Temperature (°C)
0
100
200
300
400
500
600
700
800
900
20
40
60
80
100

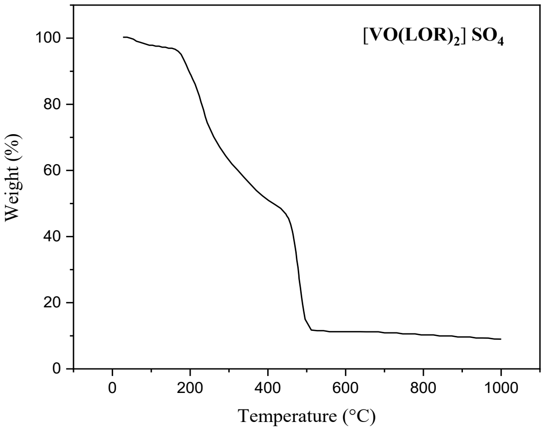
[VO(LOR)2] SO4
Weight (%)
Temperature (°C)
0
200
400
600
800
1000
0
20
40
60
80
100

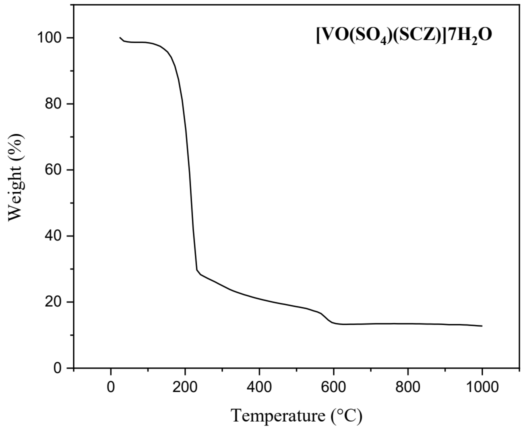

Figure S4 The TGA curves of synthesized oxidovanadium(IV) complex.

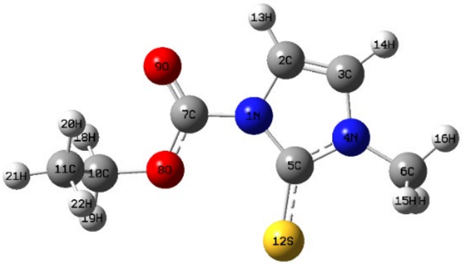

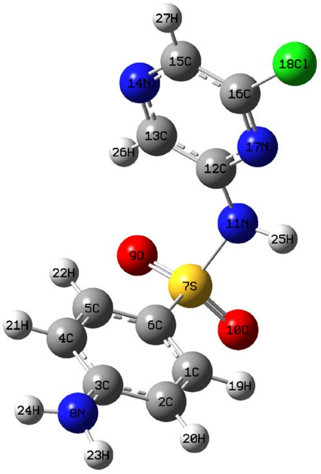

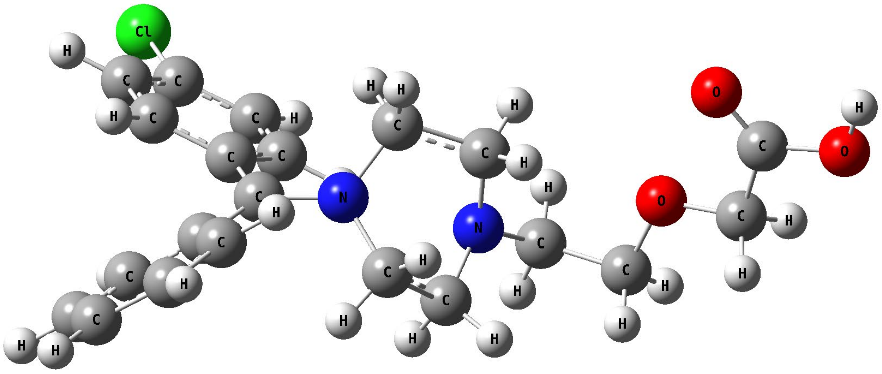

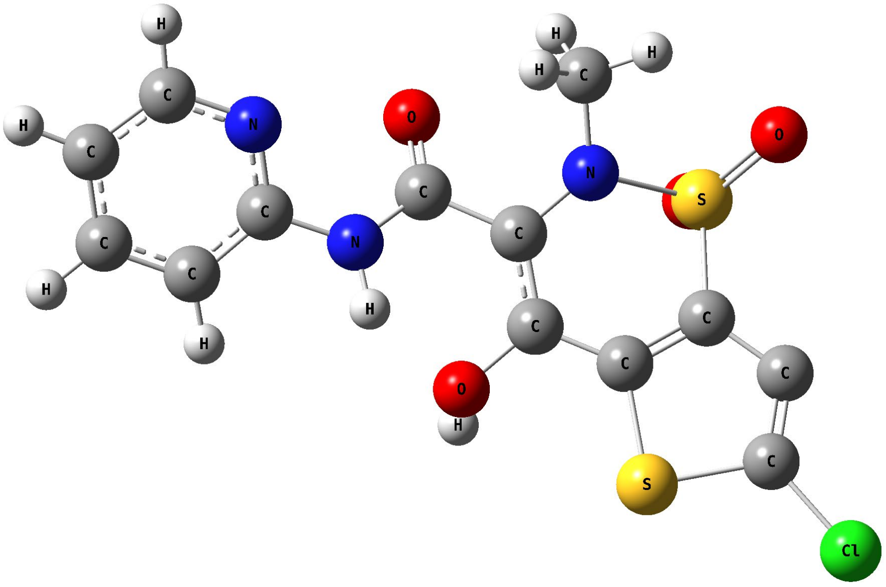

Figure S5 The optimized geometry of the free ligands gas phase using DFT method LANL2DZ basis sets.

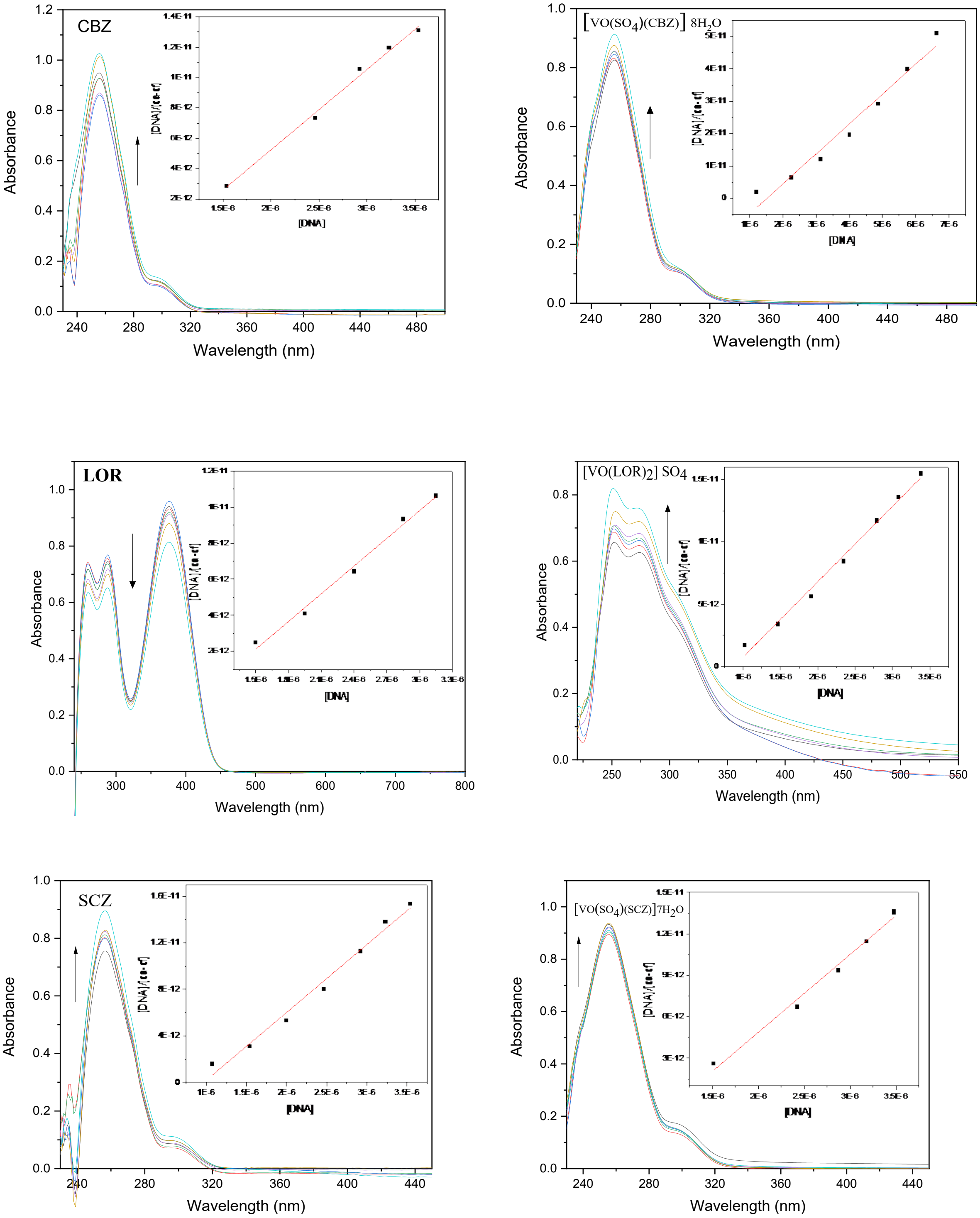

Figure S6 Absorption spectra of free ligands and their complex in the presence of increasing DNA concentration.
